# Cuprous Ions Binding to Cytosine and Guanine Oligonucleotides

**DOI:** 10.1002/cbic.70499

**Published:** 2026-08-09

**Authors:** Joana Pereira, Mathilde Brachet, Philippe Barthélémy, Bruno Alies

**Affiliations:** ^1^ ARNA INSERM, U1212 CNRS, UMR 5320 Univ. Bordeaux Bordeaux France

**Keywords:** copper complexes, cuprous ions, oligonucleotides, redox, spectroscopies

## Abstract

Using a set of spectroscopic experiments, we investigated the binding of Cu^2+^ and Cu^+^ to a comprehensive series of nucleic acid‐related molecules, ranging from individual nucleobases to oligonucleotides up to six nucleotides in length. We demonstrate the selective binding of Cu^+^ to cytosine‐ and guanine‐containing oligonucleotides, as evidenced by their ability to compete with ferrozine for Cu^+^. The capacity to inhibit ascorbate oxidation, through effective sequestration catalytically active copper, increases with the number of cytosine residues in the oligonucleotides and is associated with the formation of a 1:1 Cu^+^‐oligonucleotide complex accompanied by a pronounced conformational change. In contrast, guanine‐rich oligonucleotides exhibit the opposite behavior: they form Cu^+^ G‐quadruplex structures that permit copper redox cycling. These findings highlight an unexpected binding of copper ions to nucleic acids in inert or redox‐active modes and thus expand its potential roles within cells through its direct interaction with DNA or RNA.

## Graphical abstract

Using a comprehensive set of nucleic acid‐related molecules, from individual nucleobases to oligonucleotides, we demonstrate the selective binding of Cu^+^ to cytosine‐containing oligonucleotides. Contrary to guanine‐containing oligonucleotides, cytosine‐rich oligonucleotides form a Cu^+^‐complex with a clear near‐UV signature and are able to stop redox cycling of copper ions. These findings highlight an unexpected binding of Cu^+^ to nucleic acids in inert or redox‐active modes.

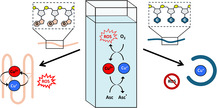

## 1 Introduction

**FIGURE 1:**
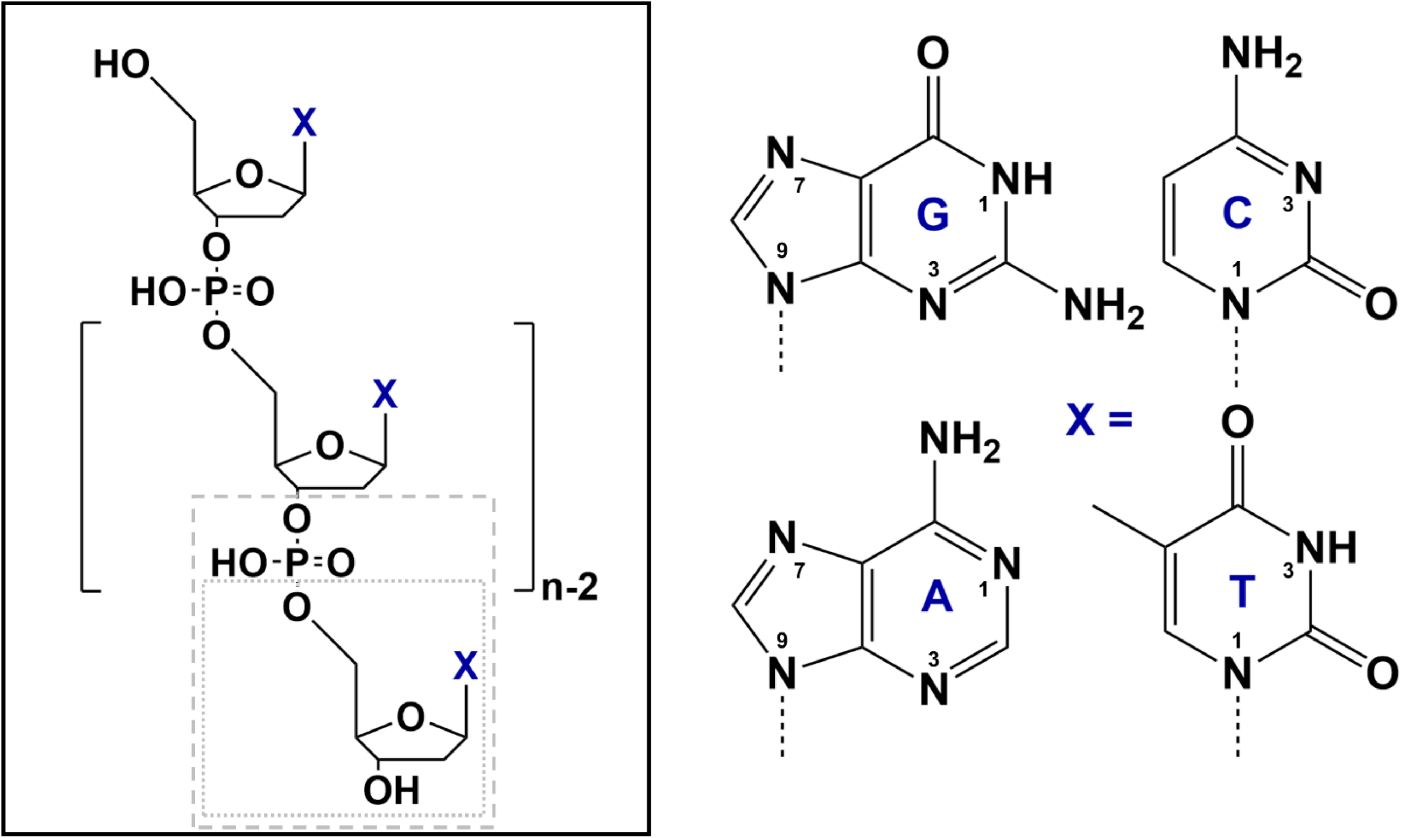
Schematic representation of studied molecules. Represented by a blue X, nucleobases (G for guanine, C for cytosine, A for adenine, and T for thymine); in the light gray dotted box, nucleosides; in the light gray dashed box, nucleotides; and in the black box, oligonucleotides X_*n*_ with *n* = 2–6.

Copper is an essential element to living organisms, involved in a multitude of physiological processes [1]. It acts primarily as a cofactor for numerous enzymes called cuproenzymes, enabling vital biochemical reactions [2, 3]. In cellular energy production, copper ions (Cu) are essential components of cytochrome c oxidase [1, 2]. To protect organisms against deleterious free radicals, copper–zinc superoxide dismutase is dependent on Cu [2], and in iron metabolism, Cu is crucial for iron absorption and hemoglobin synthesis [1]. With accessible redox potential, Cu^2+^ can be reduced to Cu^+^ biologically by physiological reductants, which gives it excellent electron transport properties, that are essential for those enzymatic reactions [4]. Although vital, excess of Cu can have harmful effects and become toxic, mainly due to its redox‐active nature, which allows it to catalyze the production of reactive oxygen species (ROS) [5]. ROS damage cell components and eventually cause cell death [1]. In Wilson’s genetic disease, accumulation of Cu is caused by a nonfunctional protein involved in Cu excretion [6]. In Alzheimer’s disease, Cu is involved in the production of oxidative damage to neurons [7], and high extracellular Cu concentrations are found in amyloid plaques [5]. In addition to ROS generation, Cu can be deleterious through its binding that can induce peptide/protein misfolding and promote aggregation [8, 9]. Due to its ambivalent nature, the interaction of Cu with DNA and other biomolecules has been extensively studied. However, for decades, the vast majority of these investigations focused exclusively on the stable Cu^2+^ and not Cu^+^ [10, 14]. To our best knowledge, there is very little scientific publications reporting behavior of Cu^+^ toward nucleic acid and DNA‐related molecules. This gap of the knowledge is very likely due to inherent difficulties to work with unstable Cu^+^. Indeed, Cu^+^ can be readily reoxidized to Cu^2+^ by dissolved dioxygen in aqueous solution, disproportionates to Cu^2+^ and Cu^0^, or precipitates into cuprous oxide (Cu_2_O). With the use of a glove box, it is now easier to overcome these limitations. However, since this instrumentation is not available in all laboratories, other methods have been developed. For example, a foundational study by Prütz [15], which drew on the work of Minchenkova and Ivanov [16], circumvented the stability issue by utilizing techniques such as pulsed and gamma radiolysis to generate Cu^+^ and observe its kinetic and structural effects on DNA and its models. In this present work, we will use a Cu^+^ stock solution stabilized by acetonitrile, and the challenges associated with its instability in solution will be overcome by first deoxygenating very carefully each solution with argon. With a limited set of techniques, mainly UV–vis, fluorescence, and circular dichroism (CD), and using minimum materials, this study aims to investigate Cu binding to nucleobases, nucleosides, nucleotides, and short‐length oligonucleotides (Figure 1) to better understand the interaction of Cu^2+^ and Cu^+^ with a comprehensive set of nucleic acid‐related molecules. The goal is to assess how a gradual increase in structural complexity and degree of organization influences molecules behavior toward Cu. Nucleobases, nucleosides, and nucleotides constitute the building blocks, while oligonucleotides allow to explore the emergence of collective behaviors related to sequence and secondary structure. The study is limited to oligonucleotides of up to six bases. Sequences of this size already cover a wide range of behaviors, from very short, loosely structured oligonucleotides to systems capable of adopting specific secondary conformations, notably G‐quadruplex‐type structures. The ability of Cu to bind to these molecules will be first assessed by a simple UV–vis experiment consisting of monitoring the depletion of ascorbate (Asc) in the presence of Cu and the tested molecules [17, 18]. We will then evaluate the specific affinity of nucleic acids for Cu^+^ or Cu^2+^ via competition experiments with two chelators (ferrozine and calcein, respectively). Finally, the effect of Cu addition on oligonucleotides will be assessed by CD.

## 2 Results and Discussion

### 2.1 Oligonucleotides Synthesis and Purity

All oligonucleotides used in this study were synthesized and purified as described in the Supporting Information. Their LC–MS profiles are presented in Figure S1. The purity of the different oligonucleotides was determined by chromatographic peak integration and was estimated at 93% for A_2_, 86% for A_3_, 73% for A_4_, 88% for A_5_, 61% for A_6_, 89% for C_2_, 81% for C_3_, 87% for C_5_, 72% for C_6_, 96% for T_2_, 95% for T_3_, 87% for T_4_, 89% for T_5_, 93% for T_6_, 74% for G_2_, 82% for G_3_, 89% for G_4_, 86% for G_5_, and 86% for G_6_. LC–MS analysis could not be performed for C_4_ due to insufficient remaining sample quantity. The presence of peak shoulders may result from oligonucleotide organization phenomena, including secondary structure formation, favored under the low‐temperature conditions (30 °C) and high‐ionic strength of the mobile phases.

### 2.2 Ascorbate Consumption Experiments

**FIGURE 2:**
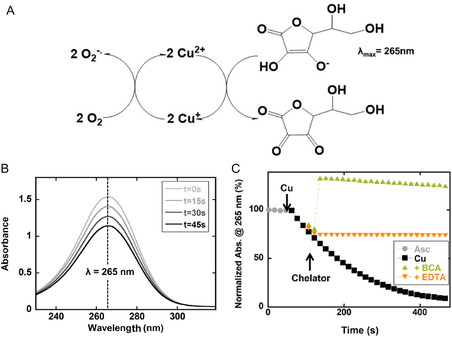
(A) Schematic representation of ROS production by Cu in the presence of Asc. (B) Absorbance of Asc in the presence of Cu over time. (C) Normalized absorbance measured at *λ* = 265 nm over time. Kinetic of Asc consumption in the absence (gray circles) or in the presence of Cu added at 60 s (black squares) with subsequent addition at 120 s of Cu^2+^ chelator EDTA (orange down triangles) or Cu^+^ chelator BCA (green up triangles). Conditions: [HEPES] = 50 mM pH 7.4; [Asc] = 100 µM; [CuSO_4_] = 10 µM; and [BCA] = [EDTA] = 22 µM.

The ability of nucleic acid and related molecules to prevent Asc oxidation by coordinating Cu^+^ or Cu^2+^ was investigated by UV–vis spectroscopy. Indeed, the presence of loosely bound Cu dramatically catalyzes the oxidation of Asc by dissolved dioxygen (Figure 2A). The kinetic rate of Asc consumption can be easily followed as the Asc shows a characteristic UV band at 265 nm (Figure 2B). In the absence of Cu, the consumption of Asc (initial concentration: 100 µM) is very slow, illustrated by a constant absorbance over time (Figure 2C, gray circles). The addition of Cu (Figure 2C, black squares) catalyzes the oxidation of Asc, as indicated by a rapid drop in absorbance (Figure 2B). If a strong Cu^2+^ chelator such as ethylene diamine‐tetra‐acetic acid (EDTA) (Figure 2C, orange down triangles) or a strong Cu^+^ chelator such as bicinchoninic acid (BCA) (Figure 2C, green up triangles) is added, Cu is tightly bound and Asc then ceases to be consumed, maintaining its constant absorbance at 265 nm [19]. The rapid and significant increase in absorbance at 265 nm after adding BCA is directly due to the fact that BCA itself absorbs strongly at 265 nm (Figure S2).

First, we tested the ability of nucleobases, nucleosides, and nucleotides to avoid the redox cycling of Cu. In the presence of these molecules, Asc is quickly consumed, except for adenine and guanine (Figure S3). Contrary to a former study, which showed that nucleosides and nucleotides, added in very large excess (up to 1000‐fold), are capable of inhibiting the Cu redox cycle [20], we demonstrate that, in our conditions, nucleosides and nucleotides have no effect on Asc consumption when they are added at a concentration of the same order of magnitude relative to Cu (Figure S4). It is interesting to note that among all these molecules tested, only the two purine bases of nucleic acids (guanine and adenine) are capable of slowing down Asc oxidation. Nitrogen N_9_ of purines bases, the most basic of N atoms, would therefore be the main atom involved in the coordination process of adenine and guanine with Cu [21, 22]. In nucleosides and nucleotides, the N_9_ is bound to the ribose and is no longer available, which could explain their lack of affinity with Cu.

**FIGURE 3:**
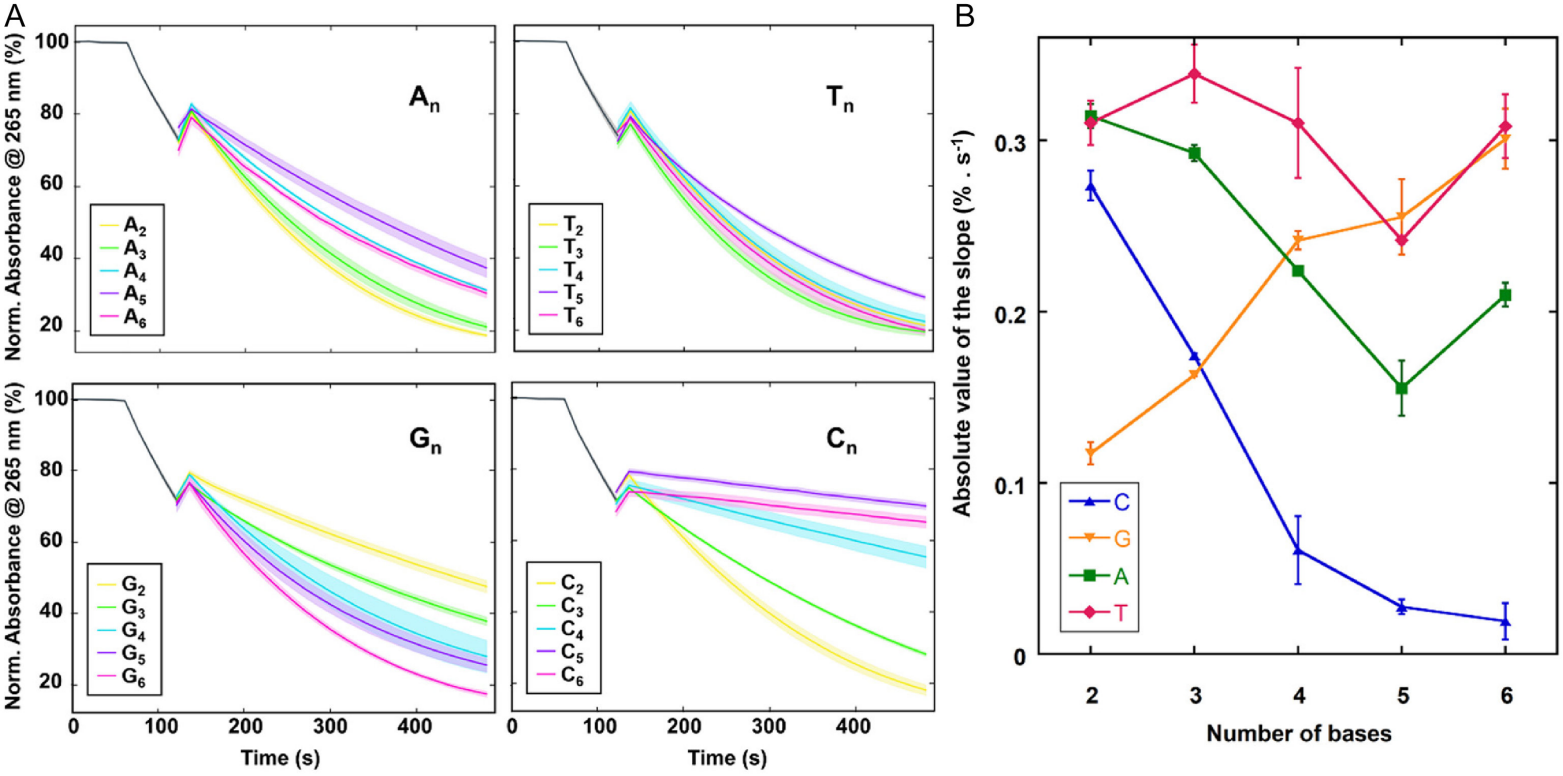
(A) Normalized absorbance measured at *λ* = 265 nm over time. Kinetic of Asc consumption in the presence of Cu (added at 60 s) with subsequent addition of short‐length oligonucleotides X_*n*_ (*n* = 2–6 bases) of adenine (top left), thymine (top right), guanine (bottom left), and cytosine (bottom right) (added at 120 s). Each bold line represents the average of three independent analyses. The width of the shaded band corresponds to the standard deviation around the mean. (B) Absolute value of the slopes of the kinetic monitoring of Asc consumption in UV–vis upon addition of Cu^2+^ and oligonucleotides as a function of the number of bases in the oligonucleotides X_*n*_. The slope is calculated between times 136 and 211 s, i.e., 6 points. Conditions: [HEPES] = 50 mM pH 7.4; [Asc] = 100 µM; [CuSO_4_] = 10 µM; and [oligonucleotides]  ×  number of bases = 20 µM.

We then tested the effect of oligonucleotide chain length (from 2–6 nucleotides) on Asc consumption (Figure 3A). For all measurements, oligonucleotide concentrations were normalized to the total nucleobase concentration. Therefore, the absorbance at 265 nm is not expected to scale directly with oligonucleotide length. First, thymine‐rich sequences have no effect on Asc consumption, clearly demonstrating that Cu has no particular affinity for thymine. In fact, carbonyl groups bind very weakly to d‐block transition divalent metal ions (M^2+^), which makes thymine less suitable for binding Cu^2+^ [12]. Cytosine‐rich oligonucleotides C_*n*_ are the most effective to prevent Asc consumption, very likely due to the binding of cytosine to Cu^2+^ or Cu^+^ via nitrogen N_3_ [13, 14, 23]. It is also interesting to note that their ability to prevent Asc consumption increases with the number of bases in its sequence, suggesting an improved sequestration of Cu likely due to an intramolecular chelation by cytosine moieties (Figure 3B). Interestingly, for sequences rich in adenine, a similar trend seems to be observed as for cytosine‐rich sequence: the more bases a sequence contains, the higher its affinity appears to be, even though these oligonucleotide sequences are not as effective in stopping Asc consumption. The ability of oligonucleotides to fold into particular structure would then modulate the coordination of Cu. Indeed, the combination of multiple binding sites, the spatial proximity of these sites allowing the formation of stabilizing chelates, and the contribution of the phosphodiester backbone [24] make oligonucleotides better chelators than nucleosides or nucleotides alone [13]. For guanine‐rich molecules, the opposite phenomenon is observed: the shortest sequences are those that best stop Asc consumption. The ability of sufficiently guanine‐rich molecules to form secondary structures such as G‐quartets via hydrogen bonds could explain this trend. Indeed, according to the literature, guanine atoms of G_*n*_ involved in the bond with Cu^2+^ are nitrogen N_7_ and oxygen O_6_ [14, 21, 22]. Thus, atoms normally involved in copper coordination would then be involved in the Hoogsteen bonds, to form the G‐quadruplex structures [25].

### 2.3 Competition Studies

**FIGURE 4:**
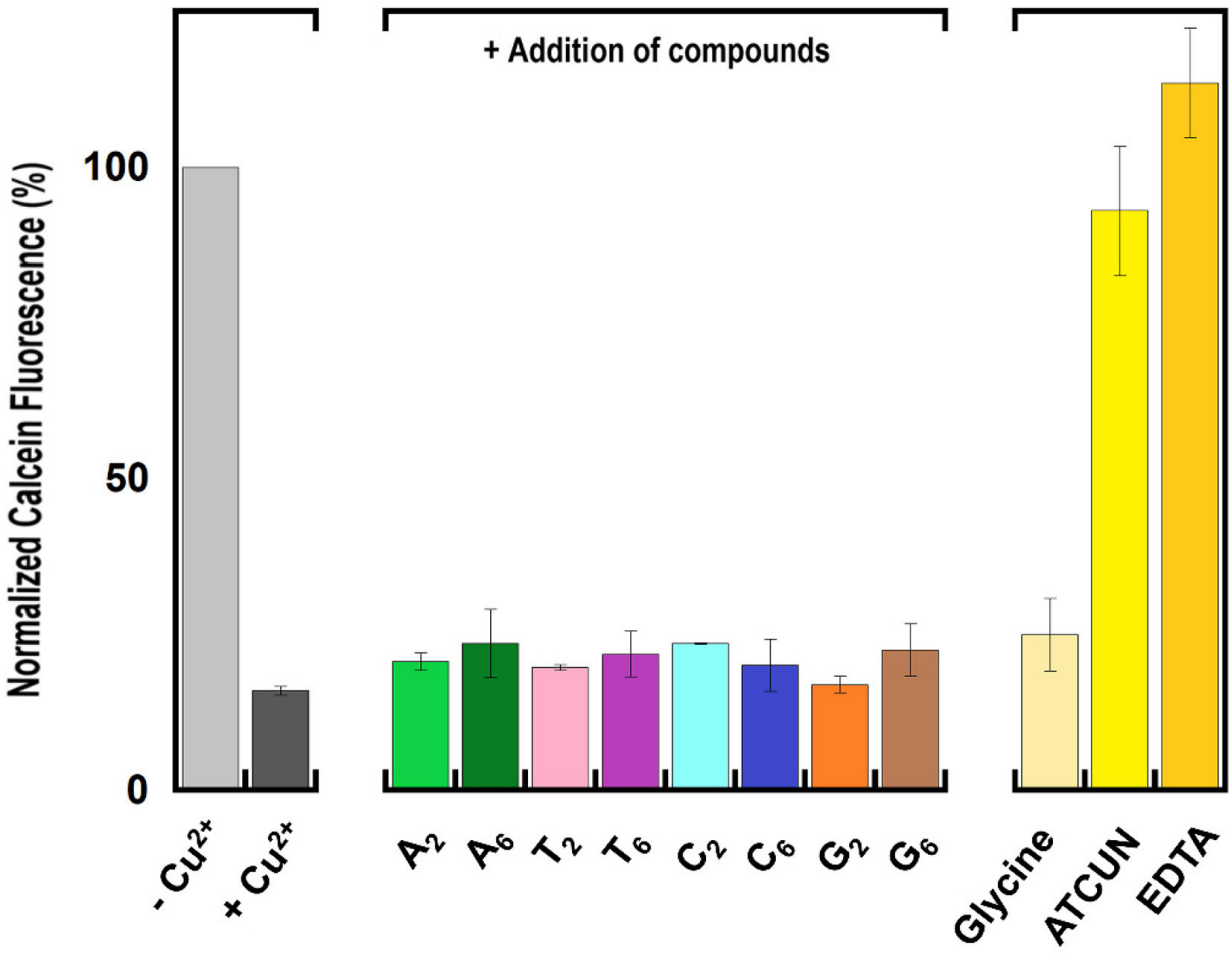
Normalized fluorescence of calcein in the absence or in the presence of Cu^2+^ (gray bars) and with addition of several compounds (colored bars). Conditions: [HEPES] = 50 mM pH 7.4; [calcein] = 1 μM; [CuSO_4_] = 0 or 1 μM; [oligonucleotides] × number of bases = 100 μM; [Cu^2+^ chelators] = 100 µM.

To understand how some of the oligonucleotides are able to stop Asc consumption, competition studies were carried out with Cu^2+^ and Cu^+^ chelators. First, we harnessed the characteristics of calcein, a fluorescent molecule with chelating properties. Fluorescence emission from calcein is observed at a physiological pH of 7.4; however, the presence of various metal cations, such as Cu^2+^, results in pronounced quenching of its fluorescence, a phenomenon often exploited for metal ion detection [26]. The ability of a molecule to compete with calcein for Cu^2+^ is reflected by the recovery of the calcein fluorescence upon addition of this molecule. Therefore, the intensity of the fluorescence of calcein upon addition of a molecule shows the relative affinity of the added molecule for Cu^2+^. In Figure 4, different Cu^2+^ chelators with different affinities illustrate this phenomenon. EDTA has the highest affinity for Cu^2+^ (log *K* = 19.19) [27], followed by the amino‐terminal copper and nickel (ATCUN) binding motif peptide (log *K* ~ 14) [28] and finally glycine (log *K* = 8.28) [29]. EDTA, ATCUN motif, and glycine were employed as reference ligands to qualitatively compare Cu^2+^ binding affinity and competition behavior. Oligonucleotides X_*n*_ have few effects on fluorescence, suggesting that they are not sufficiently affined with Cu^2+^ to sequester it from calcein. The findings from the competition assay indicate that the metal–ligand complex stabilities of the oligonucleotides are all relatively similar and that oligonucleotides have an affinity constant equal or lower than glycine. However, in contrast to Asc consumption, C_2_ was found to be more effective than C_6_ at recovering Cu^2+^ from calcein, while G_6_ similarly exhibited greater efficacy than G_2_. These observations raise the question of whether the affinity of oligonucleotides for Cu^2+^ influences the inhibition of Asc consumption. Given that glycine and oligonucleotides have comparable affinity constants, the experiment to stop Asc consumption was also performed in the presence of 20 µM glycine (Figure S5). It should be noted that glycine has no absorbance at 265 nm, so no increase in absorbance was observed when it was added at 120 s. In the presence of glycine, Cu catalyzes the oxidation of Asc with a consumption rate of 0.347 ± 0.058%/s (absolute value), which makes it comparable in terms of efficiency to short‐thymine sequences. Thus, despite the fact that oligonucleotides have a lower or comparable affinity than glycine for Cu^2+^, the impact on Asc consumption varies considerably, with oligonucleotides demonstrating greater inhibition of the redox cycle than glycine. It is therefore relevant to evaluate the influence of affinity for Cu^+^ on their ability to slow down Asc consumption.

**FIGURE 5:**
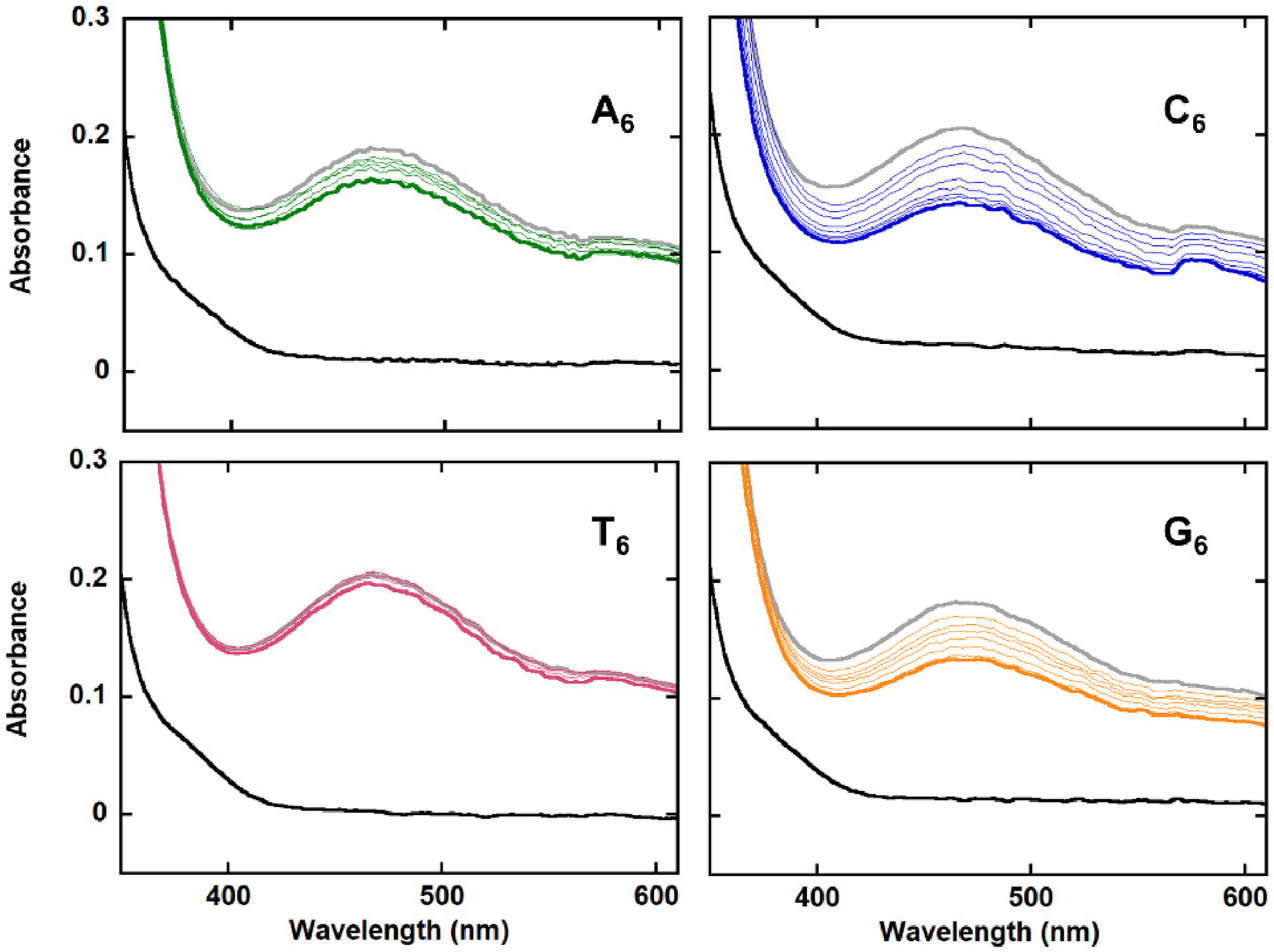
UV–vis spectra of successive additions of oligonucleotides A_6_ (top left), C_6_ (top right), T_6_ (bottom left), and G_6_ (bottom right) to a solution of [Cu(Fz)_2_]. Conditions: [HEPES] = 50 mM pH 7.4; [Fz] = 110 μM; and [Cu^+^] = 55 μM. The black curve corresponds to [Cu^+^] = 0 μM. The gray curve corresponds to the formation of the complex after adding Cu^+^. The colored curves correspond to the addition of X_*n*_ from 3.8 µM up to 110 µM (bold curve). For details on the specific concentration increments, see Experimental Details in the Supporting Information.

Competition experiments with ferrozine were then carried out. Ferrozine forms a 2:1 complex with Cu^+^ (log *β*_2_ = 11.6), characterized by a band in the visible range at 470 nm [30]. In Figure 5, the effect of successive addition of the 6‐bases long oligonucleotides X_6_ to a solution of [Cu(Fz)_2_] is shown. Addition of C_6_ leads to a significant decrease in the 470 nm absorption band characteristic of the [Cu(Fz)_2_] complex, demonstrating its affinity for Cu^+^ (log *K* = 6.5), for calculation details refer to Experimental Details in the Supporting Information. The results indicate that G_6_ is also effective at sequestering Cu^+^ (log *K* = 5.9). However, its effectiveness appears to be lower than that of C_6_ but higher than that of A_6_ (log *K* = 5.2). T_6_, on the other hand, does not appear to have any particular affinity for Cu^+^.

**FIGURE 6:**
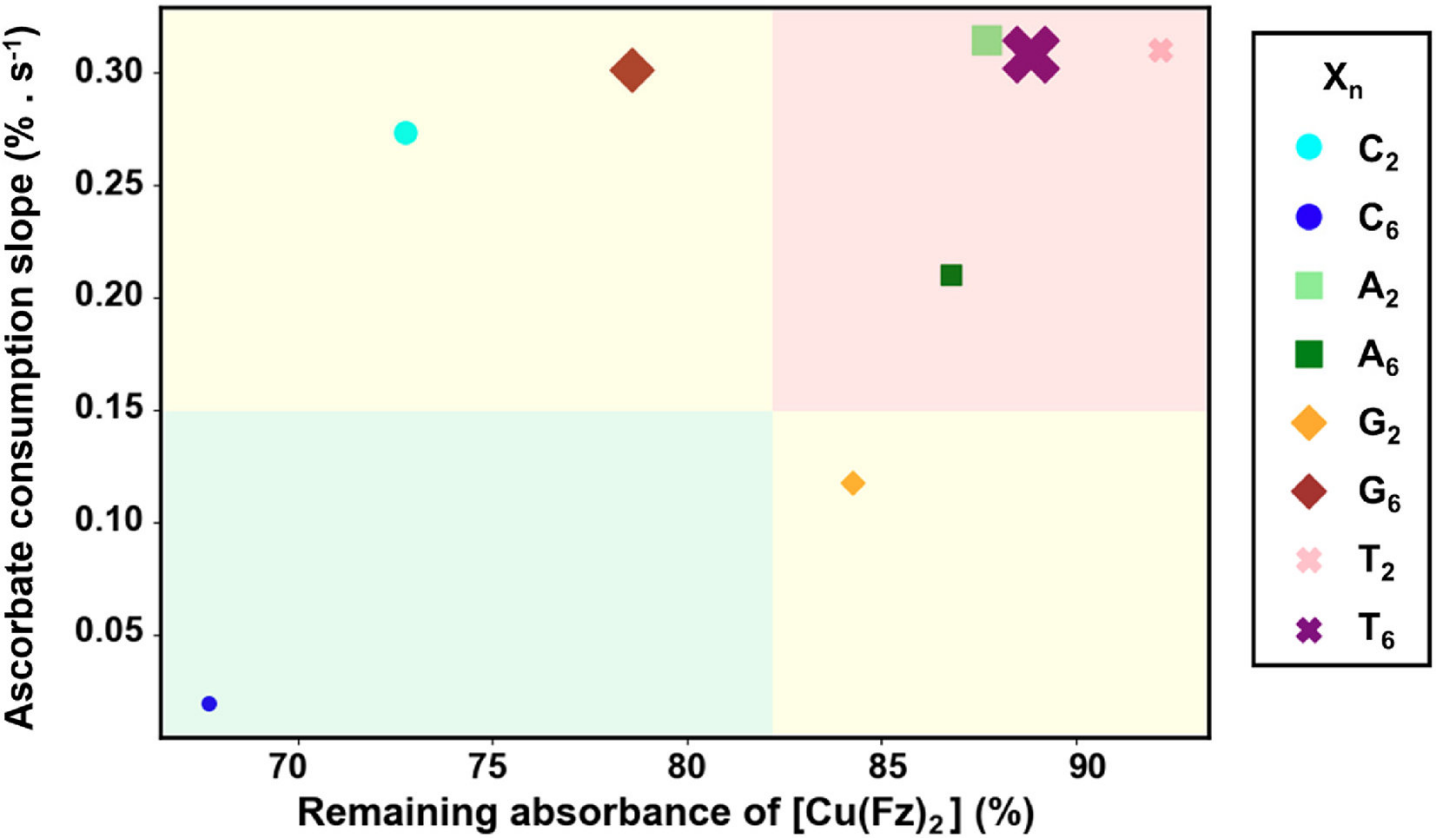
Oligonucleotides are represented by points whose coordinates are the respective values of the percentage of absorbance remaining in the [Cu(Fz)_2_] complex after adding 660 µM of nucleobases of X_*n*_ at 470 nm and the slope of Asc consumption. Points in the green zone represent molecules capable of slowing down Asc consumption and recovering Cu^+^ to ferrozine. The red area represents molecules that are unable to stop or recover Cu. The orange areas are those with one of the two successful assays. The size of the icon represents the standard deviation by on three independent experiments. The limits of the different colored areas were defined arbitrarily for visualization purposes and do not correspond to strict physicochemical thresholds.

The results are broadly in line with those obtained in Asc consumption assay. Oligonucleotides that slow down Asc oxidation are also able to bind Cu^+^ in ferrozine competition experiments. In the same way, most of the oligonucleotides with no effect on Asc consumption do not lower this absorption band of the Cu^+^‐ferrozine complex (Figure 6). Interestingly, G_6_ seems capable of binding to Cu^+^ and decreasing the absorption band but fails to slow down Asc consumption (Figures 5 and 6), suggesting the formation of a redox‐active complex between Cu and the oligonucleotide, thereby allowing the reduction and the oxidation of Cu during Asc consumption experiments.

### 2.4 Titration

**FIGURE 7:**
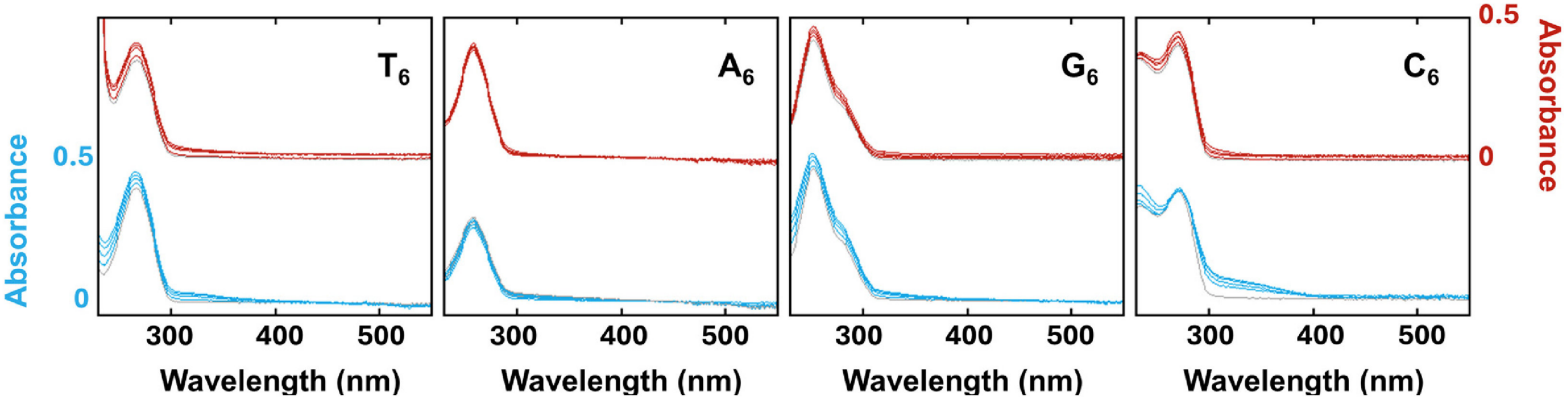
UV–vis spectra of oligonucleotides T_6_; A_6_; G_6;_ and C_6_ (from left to right, gray) and successive additions of Cu^2+^ (scale on right axis, red) and Cu^+^ (scale on left axis, blue). Conditions: [HEPES] = 50 mM pH 7.4; [T_6_] = 6 µM; [A_6_] = 3.5 µM; [G_6_] = 6 µM; [C_6_] = 8 µM; [Cu^2+^] = 0–20 µM; and [Cu^+^] = 0–20 µM.

Nucleobases, nucleosides, nucleotides, and oligonucleotides X_2_ and X_6_ were titrated with Cu^2+^ and Cu^+^ solutions. The UV spectra of all molecules are included in the Supporting Information (Figure S6 for adenine family, Figure S7 for thymine family, Figure S8 for guanine family, and Figure S9 for cytosine family) with the exception of T_6_, A_6_, G_6_, and C_6_, which are presented in Figure 7. As expected, titration of the molecules with Cu^2+^ resulted in a uniform increase in absorbance intensity, with no significant spectral changes observed, suggesting weak or nonspecific interactions. The only molecules that behaves differently are the nucleobases adenine and guanine, which shows a slight bathochromic shift upon Cu^2+^ addition (likely due to availability of atom N_9_ which is no longer available in nucleosides, nucleotides, or oligonucleotides, as involved in glycosidic bond to the ribose). The results of the Cu^2+^ titration with oligonucleotides are consistent with the results previously obtained using calcein competition. Similarly, in the presence of Cu^+^, a change in the UV spectrum of nucleobase adenine is observed, indicating a strong interaction between the base and the metal cation (Figure S6). The titration of the other molecules, including T_6_, A_6_, and G_6_ with Cu^+^, resulted also in a small uniform increase in absorbance intensity without spectral changes, suggesting for the oligonucleotides T_6_ and A_6_, an absence of a specific interaction, and for G_6_, a nonperturbing binding that does not alter the structure and the electronic transitions of the molecule. However, titration of C_6_ with Cu^+^ led to the emergence of a new large absorption band around 315 nm, strongly indicating the formation of a unique type of Cu^+^ complex, likely with a 1:1 stoichiometry (Figure S10). It is worth noting that the C_6_ affinity for Cu^+^ determined by direct titration (log *K* = 6.3, Figure S10) is in good agreement with affinity obtained from ferrozine competition experiments (log *K* = 6.5, Figure 5). This absorption band can also be observed in the spectrum of Asc consumption experiment in the presence of Cu and C_6_ (Figure S11). The presence of this band suggests that the ability of C_6_ to halt the consumption of Asc comes from its capacity to chelate Cu^+^ and form a complex stable enough to prevent the reoxidation of Cu^+^ to Cu^2+^ in the presence of oxygen.

### 2.5 Circular Dichroism

To complement the titration analyses, CD experiments were then carried out to compare the signal of oligonucleotides alone and in the presence of both redox states of Cu at stoichiometry 1:1. The objective is to investigate the structure of oligonucleotides X_2_ and X_6_ in the presence of Cu^+^ and Cu^2+^ in order to address the possible change in their structures, which would not have been observed in the UV–vis experiments. A range of information can be extracted from these results. First, it has been demonstrated that in the presence of Cu^2+^ and Cu^+^, the CD spectra of thymine‐ and adenine‐rich molecules are identical to the spectra of the molecules alone (Figure S12). The presence of Cu therefore does not alter the overall structure of the oligonucleotides. These results are consistent with the results of previous experiments, allowing us to deduce that A_2_, A_6_, T_2_, and T_6_ have little affinity and do not form complexes with Cu^+^, as mentioned in a former study [15]. Regarding oligonucleotides composed of cytosine, their CD spectra are very similar to their spectrum in the presence of Cu^2+^. However, in the presence of Cu^+^, dramatically changes in the CD spectrum are observed.

**FIGURE 8:**
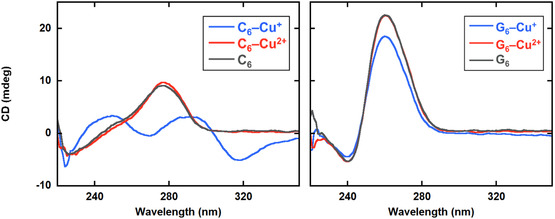
CD spectra of oligonucleotides C_6_ (left) and G_6_ (right) in the absence and in the presence of Cu^+^ and Cu^2+^ at stoichiometry 1:1. Conditions: [HEPES] = 50 mM pH 7.4; [C_6_] = [Cu^+^] = [Cu^2+^] = 8 µM; and [G_6_] = [Cu^+^] = [Cu^2+^] = 5 µM.

For C_2_ (Figure S13), the band at 280 nm is replaced by a positive band at 250 nm. This indicates that their three‐dimensional structure has been modified in the presence of Cu^+^. For C_6_ (Figure 8), the positive band at 280 nm disappears, giving way to a negative band at 320 nm. With increasing amounts of Cu^+^, the spectral changes were progressively observed until reaching a plateau at a 1:1 Cu^+^/C_6_ ratio, confirming the formation of a 1:1 complex with Cu^+^ (Figure S14). Figure S15 shows control experiments of the UV and CD spectra of C_6_ under an argon atmosphere with acetonitrile addition, clearly illustrating that the changes observed before are only due to Cu^+^ and not to the low amount of acetonitrile added with Cu^+^ addition. The results of these various analyses carried out on cytosine‐rich molecules suggest that Cu^2+^ prefers to remain solvated in biological aqueous media (hydration enthalpy = −2100 kJ/mol), whereas an interaction between Cu^+^, which is less hydrated (hydration enthalpy = −593 kJ/mol), and cytosine occurs, as evidenced by the modification of the CD spectra [31]. We suggest that the coordination site of the Cu^+^‐C_6_ complex consists of cytosine‐Cu^+^‐cytosine, where Cu^+^ is bound to the nitrogen atoms N_3_ from two cytosines, analogous to the Cu^+^–i‐motif DNA structure and to Ag^+^ interacting with cytosines [31, 32]. The CD spectra of G_2_ alone and in the presence of Cu^2+^ are very similar (Figure S13). However, a small positive CD signal is observed near 240 nm, only in the presence of Cu^+^, which could be interpreted as an interaction between the two species. Regarding G_6_, its CD spectrum is characterized by a positive peak at 260 nm and a negative peak at 240 nm. This dichroic signature corresponds to the presence of parallel G‐quadruplexes [33]. This result supports our hypothesis regarding the ability of G_*n*_ oligonucleotides to form G‐quadruplexes in HEPES buffer. These characteristic peaks are also observed in the presence of Cu. With Cu^2+^, the CD spectra are similar. However, when Cu^+^ is added, the positive peak at 260 nm is less intense. This observation could be explained by the fact that G_6_ and Cu^+^ interact without modifying the G‐quadruplex structure adopted by G_6_, but by destabilizing it, hence the decrease in intensity. Thus, G_6_ may rearrange itself specifically to bind Cu^+^ while retaining its G‐quadruplex identity, forming a complex. To support this hypothesis, CD analysis of the G_5_ oligonucleotide, which is also capable of forming a G‐quadruplex structure, was performed in the presence of different concentrations of Cu^+^ (Fig. S16). As the amount of Cu^+^ increases, the intensity of the G‐quadruplex decreases. The ability of G_6_ to retrieve Cu^+^ from ferrozine but not to prevent the oxidation of Asc strongly suggests that this complex allows the redox change by binding the different oxidation states of Cu. This interpretation is supported by recent papers of Sahu et al. [34, 35] that show that Cu^+^ can induce the formation of highly stable G‐quartets from guanosine, without K^+^ or Na^+^ and that these G‐quartets selfassemble to form supramolecular polymers. Furthermore, their results suggest that this supramolecular assembly is labile and allows Cu^+^ to oxidize to Cu^2+^ without immediately destroying the structure but which can reversibly dismantle it in response to this oxidation.

These changes in CD and UV spectra effectively support our results that cytosine‐rich molecules have an affinity for Cu^+^ which will cause a structural change in the oligonucleotides, while guanine‐rich molecules can interact with Cu^+^ by keeping the G‐quadruplex structures.

## 3 Conclusion

In this study, the interaction of DNA‐related molecules with Cu was investigated using simple spectroscopic methods with a focus on small oligonucleotide. We demonstrate that oligonucleotides composed of cytosine or guanine have a strong affinity for Cu^+^. Cytosine‐rich oligonucleotides appear to form an inert complex with Cu^+^, while guanine‐rich oligonucleotides, structured in the form of G‐quadruplexes, enable the redox cycle of Cu. The implications of this research are important for better understanding the interaction of DNA/RNA with Cu, which is present in the body. The uptake of Cu inside cell is mediated by CTR1 protein [36]. Once inside the cell, free Cu^+^ is immediately sequestered by chaperones. Nevertheless, each human cell contains approximately two meters of DNA coiled and compacted within its nucleus [37] and about 6 pg and 20 pg of DNA and RNA materials, respectively [38]. Based on these numbers, concentrations of nucleobases within nucleic acid polymers are about 50 mM both in the cytoplasm and nucleus. This enormous concentration and a vast contact surface provide multiple potential sites for Cu^+^ binding. The presence of this element inside the cell, even at low concentrations and sequestered, can create an environment conducive to unexpected interactions with nucleic acids, which would compete with metallochaperones given their overwhelming concentration. Furthermore, the ends of human chromosomes, rich in guanine repeats, can spontaneously form G‐quadruplexes, which could regulate telomerase activity and act as a protective cap at the end of chromosomes, preventing telomere DNA from degrading [39]. However, we have shown that Cu^+^ can interact with these structures, and thus, Cu^+^ could modulate the stability of telomeric G‐quadruplexes. Moreover, with the growing interest in using nucleic acids‐based therapeutics, it is critical to expand our understanding of their chelating properties. This knowledge is essential for integrating them into potential treatments linked to copper imbalance, such as in Alzheimer’s and Wilson’s disease.

## Funding

This study was supported by Conseil National de la Recherche Scientifique (CNRS) via MITI programs (Metallo‐Mix call) and FrenchBIC.

## Conflicts of Interest

The authors declare no conflicts of interest.

## Supporting information

Supplementary Material

## Data Availability

The data that supports the findings of this study are available in the supplementary material of this article.
