# Supplementary material for: Cuprous Ions Binding to Cytosine and Guanine Oligonucleotides
Source: Chembiochem. 2026 Aug 9;27(15):e70499. doi: 10.1002/cbic.70499 (PMC13453070; doi:10.1002/cbic.70499)
Supplement: Supplementary file 1 [file CBIC-27-e70499-s001.pdf]

Supporting Information

CUPROUS IONS BINDING TO CYTOSINE AND GUANINE OLIGONUCLEOTIDES

J. Pereira, M. Brachet, Prof. P. Barthélémy, Dr B. Alies *

Experimental Details

Chemicals and Oligonucleotides

All chemicals were purchased from Sigma-Aldrich. Oligonucleotide molecules were synthesized on an automated H8 DNA synthesizer (K&A Laborgeraete, Germany) at a 1 µmol scale and deprotected in ammonia at 55°C for 4H, in the laboratory. The details of the synthesis and deprotection steps are described in ref [40]. For all experiments measurements, oligonucleotide samples were prepared by normalizing the concentration to the total nucleobase content rather than to the molar concentration of oligonucleotide strands. Thus, the concentration of each oligonucleotide solution was adjusted according to the number of nucleotide units present in the sequence in order to maintain a comparable total concentration of absorbing bases across all samples. For example, a 6-mer oligonucleotide was prepared at a concentration two times lower than a 3-mer in order to maintain an equivalent total nucleotide concentration. The Cu^{+} source originates from $Cu(MeCN)_4BF_4$ powder, which was used to prepare a high-concentration stock solution in acetonitrile. Concentration of Cu^{+} is controlled using BCA. Further dilution were made in water in order to keep the concentration of acetonitrile lower than 0.5 % v,v. The Cu^{2+} source originates from $Cu(SO_4).5H_2O$ powder, which was used to prepare a high-concentration stock solution in water. EDTA was purchased from Sigma-Aldrich and ATCUN motif peptide (with the sequence EVHH) was ordered from Bachem.

LC/MS characterization of oligonucleotides

The purity of the oligonucleotides was assessed by liquid chromatography coupled with mass spectrometry (LC/MS). The analytical method is currently being optimized and has not yet been published, therefore, detailed chromatographic and spectrometric conditions are not disclosed in this study. The purity of the oligonucleotide of interest was determined by integrating the chromatographic peaks and expressed as a relative percentage of the total integrated peak area corresponding to all detected species.

UV-Visible experiments

UV-visible absorption spectra and Asc kinetic experiments were recorded on an Agilent Cary 8454 Spectrophotometer in a 1 cm path length quartz cuvette. All measurements were carried out at 25 °C.

Samples were prepared from stock solutions of the different compounds at the concentrations needed in the experiments.

For Asc kinetic experiments, a solution of Cu^{2+} at 1 mM was prepared from a solution of $CuSO_4$ at 0.1 M. Asc was freshly prepared and diluted to 100 µM in the cuvette in 50 mM HEPES buffer, pH 7.4. Cu^{2+} was added at 10 µM concentration at 1 min. At 2 min, candidates were added. The added molecules exhibit different intrinsic absorbance intensities at 265 nm, therefore, their addition cause an increase of the absorbance that vary depending on the molecules added. The absorbance at 265 nm normalized (at t=0s) was plotted against time. The kinetic decrease of absorbance at 265 nm is due to the depletion of Asc (which absorbs λ_{max} = 265 nm, ε = 14 500 $M^{-1}.cm^{-1}$) over time.

For competition experiments with Ferrozine, a 5 mM solution of Cu^+ was prepared from a 12 mM stock solution in acetonitrile that originates from $Cu(MeCN)_4BF_4$. Concentration of Cu^+ is controlled using BCA. Ferrozine was freshly prepared in 50 mM HEPES buffer at 110 µM and degassed with argon.
The blank was first made in the cell with ultrapure water, then 200 µL of Ferrozine solution was added and degassed with argon. 2.2 µL of Cu^+ solution was added with a Hamilton syringe and the solution was mixed with the bubbles produced by the argon. Compounds were then added progressively to obtain concentrations ranging from 22.5 to 660 µM divided by the number of compound nucleotides (for A_6 and T_6: 22.5 / 55 / 110 / 330 / 550 / 660 µM; for G_6: 22.5 / 55 / 110 / 220 / 330 / 550 / 660 µM; for C_6: 22.5 / 55 / 110 / 330 / 440 / 550 / 660 µM). All the solution were previously degassed with argon before being introduced in the tightly sealed UV-vis cuvette kept under argon.

For the titration experiments, the samples were first adjusted to achieve an initial absorbance of approximately 0.30 at 260 nm, thereby ensuring comparable starting conditions across samples. Cu^{2+} was then added gradually until a final excess of Cu^{2+} corresponding to a molar ratio of 2.5 relative to the nucleic acid concentration was reached. After each addition, the solution was mixed and allowed to reach equilibrium before recording the UV-Vis spectra. For the Cu^+ titration, the samples were degassed with argon prior to the addition of Cu^+. Cu^+ was then added gradually as described with Cu^{2+} using a Hamilton syringe and the solution was mixed with the bubbles produced by the argon and allowed to reach equilibrium before recording the UV-Vis spectra.

Determination of Cu^+ binding constant with oligonucleotides X_6

To determine Cu^+ binding constant with oligonucleotides X_6, the results of the competition experiment with Ferrozine were used directly in formula (1).

$$K_{Cu(X_6)} = \frac{\beta_{CuFz_2}\,[Fz]_{free}{}^2[Cu(X_6)]}{[X_6]_{free}[CuFz_2]} \quad (1)$$

The final concentration of the $[Cu(Fz)_2]$ complex was determined based on the residual absorbance measured after the addition of the oligonucleotides, relative to the initial absorbance of the fully formed complex.

The concentration of the $[Cu(X_6)]$ complex was then calculated from the decrease in $[Cu(Fz)_2]$ concentration upon addition of the oligonucleotide, assuming a Cu/X_6 stoichiometry of 1:1.
The concentration of free ferrozine ($[Fz]_{free}$) was obtained by subtracting the concentration of bound ferrozine ($2 \times [Cu(Fz)_2]$) from the initial concentration of ferrozine.
The concentration of free X_6 ($[X_6]_{free}$) was calculated by subtracting the concentration of Cu-bound X_6 ($[Cu(X_6)]$) from the initial concentration of X_6.

Determination of Cu^+ affinity by direct titration is based on data presented on Fig. S10 left panel and on the use of the below formula (2), where $K_{Cu(C_6)}$ is the affinity of C_6 for Cu^+; $[Cu(C_6)]$ is the concentration of the complex; $[C_6]$ is the concentration of C_6 unbound to Cu^+; $[Cu]$ is the concentration Cu^+ unbound to C_6.

$$K_{Cu(C_6)} = \frac{[Cu(C_6)]}{[C_6].[Cu]} \quad (2)$$

The concentration of the $Cu(C_6)$ complex was then calculated from the increase of 315 nm band assuming a $Cu:X_6$ stoichiometry of 1:1 and a total formation of the complex at the end of the titration.
The concentration of unbound C_6 was obtained by subtracting the concentration of $Cu(C_6)$ from the initial concentration of C_6. The concentration of unbound Cu^+ was calculated by subtracting the concentration of $Cu(C_6)$ from the actual added concentration of Cu^+.

Circular Dichroism experiments

CD spectra was performed on JASCO J-1500 Circular Dichroism (optical path length = 1 cm) in quartz cuvette coupled with a Peltier device. All measurements were carried out at 25 °C. The spectra have been corrected to the water spectrum. The concentration of each oligonucleotide was determined to obtain an initial absorbance of approximately 0.30 at 260 nm. Oligonucleotides were analyzed alone, then in the presence of Cu^{2+} and Cu^+ (under argon atmosphere) at 1:1 stoichiometry.

Fluorescence experiments

Competiton experiment with Calcein was performed in 96-well plates with fluorescence measured at λ_{ex} = 495 nm and λ_{em} = 515 nm using the Infinite® M1000 Tecan microplate reader. Calcein was added at 1 µM concentration in HEPES 50 mM, pH 7.4 and its fluorescent was read. Then, 1 µM Cu^{2+} was added and fluorescence was measured after night. Then, compounds were finally added at a concentration of 100 µM divided by the number of nucleotides and the final fluorescence of Calcein in presence of Cu^{2+} and oligonucleotides was measured after 4 hours.

Reference

[40] S. Pourshahian, “Therapeutic Oligonucleotides, Impurities, Degradants, and Their Characterization by Mass Spectrometry.” *Mass Spectrometry Reviews* **40**, (2021): 75–109.

Supplementary Figures

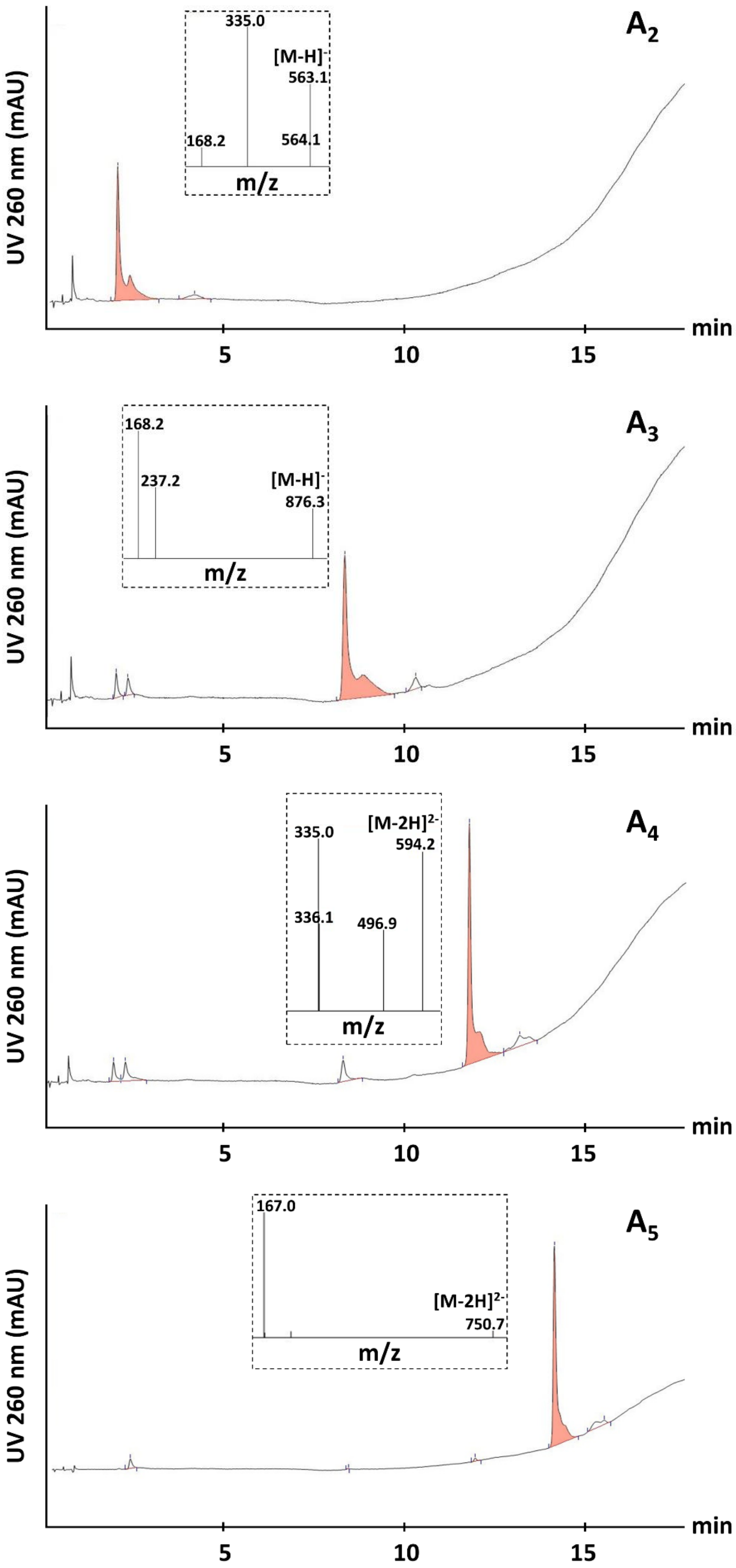

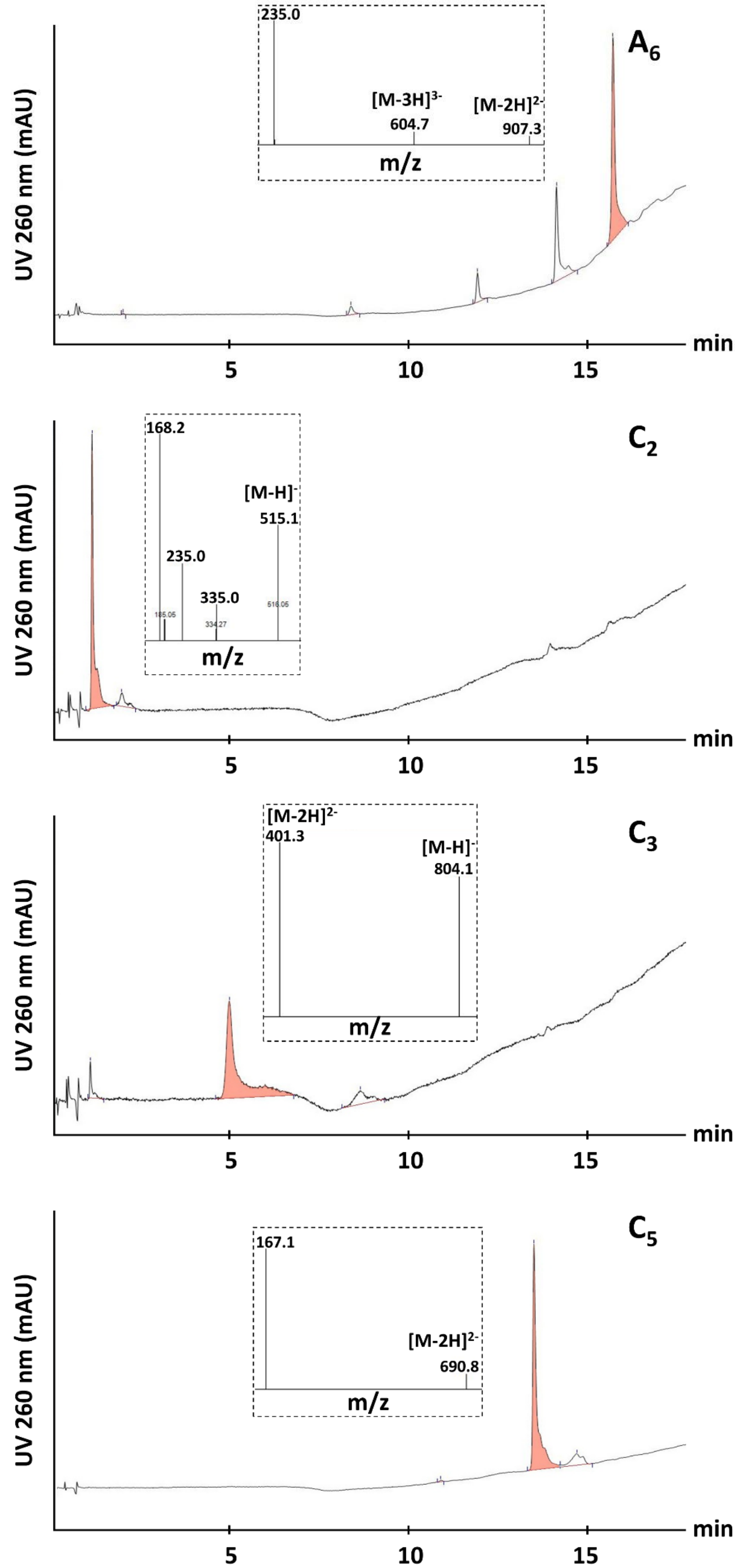

A6
UV 260 nm (mAU)
235.0
[M-3H]3-
604.7
[M-2H]2-
907.3
m/z
min
5
10
15
C2
UV 260 nm (mAU)
168.2
[M-H]-
515.1
235.0
335.0
m/z
min
5
10
15
C3
UV 260 nm (mAU)
[M-2H]2-
401.3
[M-H]-
804.1
m/z
min
5
10
15
C5
UV 260 nm (mAU)
167.1
[M-2H]2-
690.8
m/z
min
5
10
15

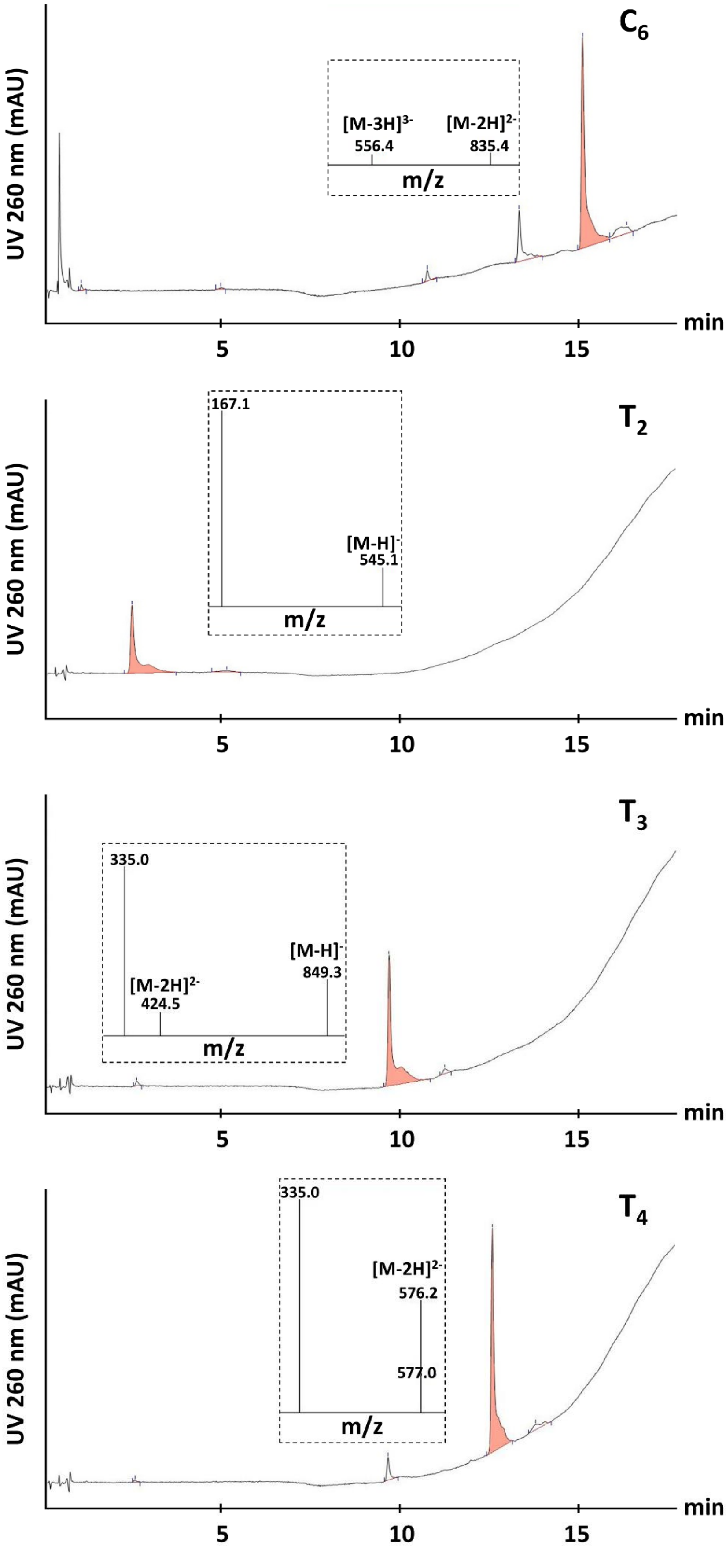
C6
UV 260 nm (mAU)
[M-3H]3-
556.4
[M-2H]2-
835.4
m/z
min
5
10
15
T2
UV 260 nm (mAU)
167.1
[M-H]-
545.1
m/z
min
5
10
15
T3
UV 260 nm (mAU)
335.0
[M-2H]2-
424.5
[M-H]-
849.3
m/z
min
5
10
15
T4
UV 260 nm (mAU)
335.0
[M-2H]2-
576.2
577.0
m/z
min
5
10
15

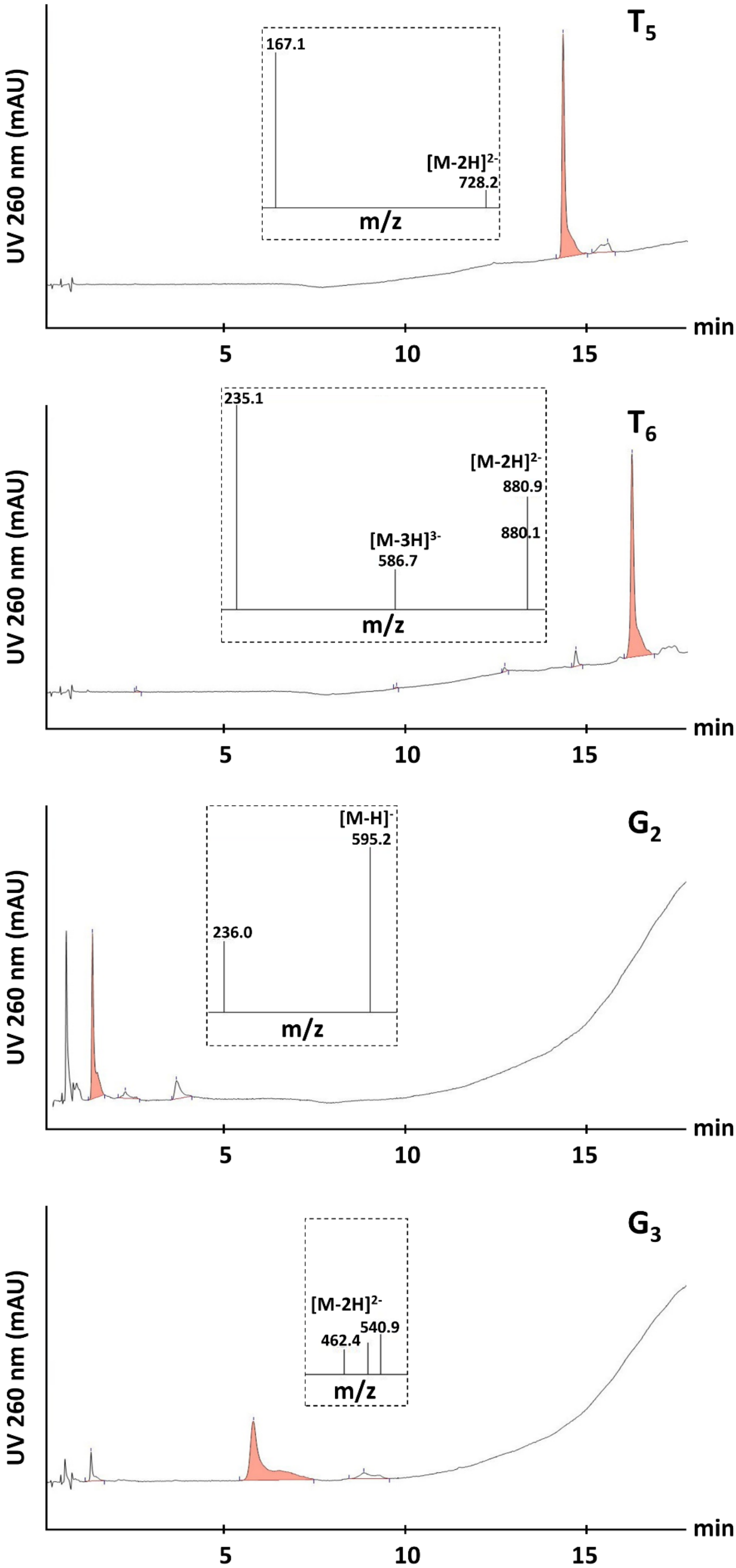
T5
UV 260 nm (mAU)
167.1
[M-2H]2-
728.2
m/z
min
5
10
15
T6
235.1
[M-2H]2-
880.9
880.1
[M-3H]3-
586.7
G2
[M-H]-
595.2
236.0
G3
[M-2H]2-
462.4
540.9

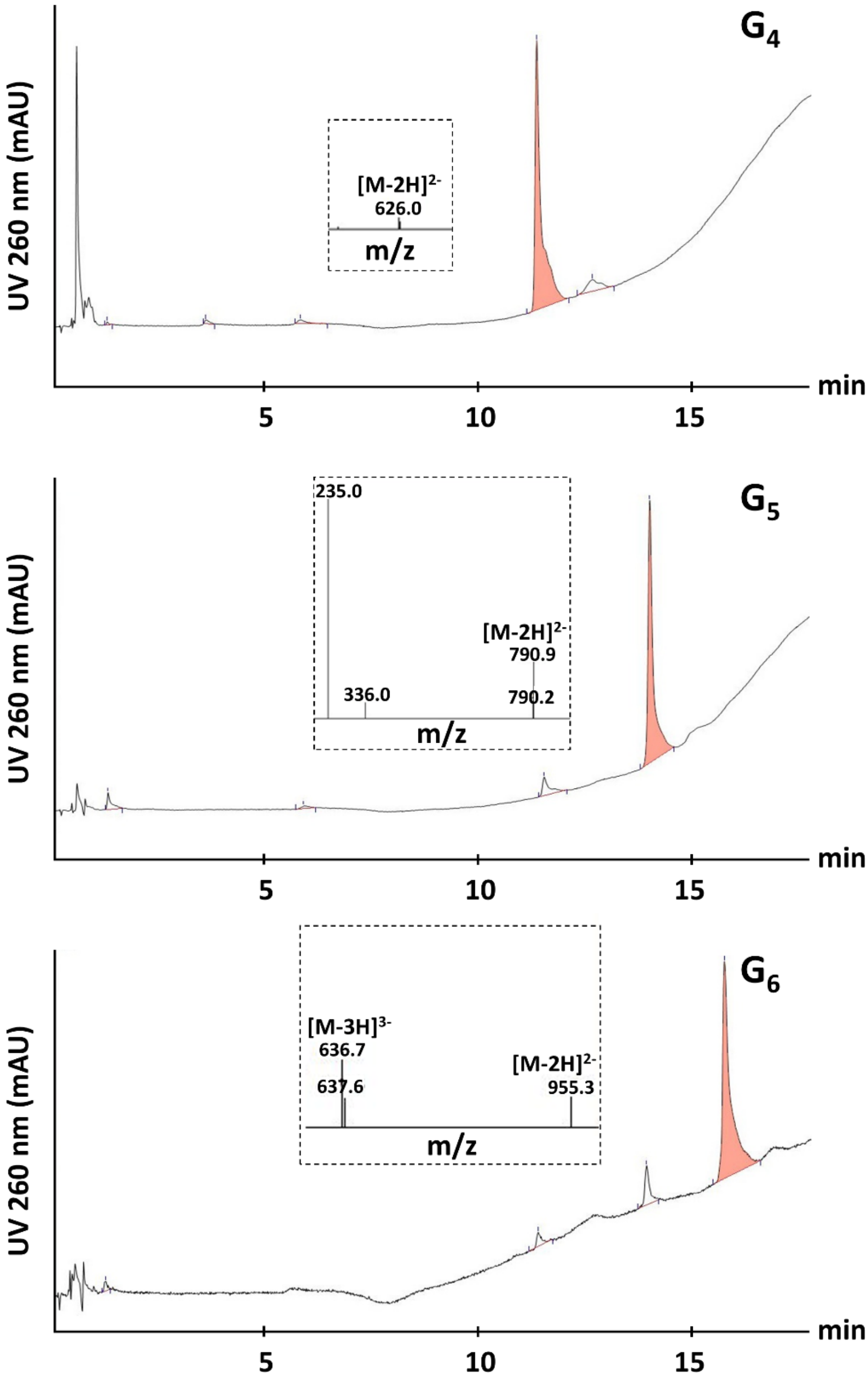

Figure S1. Representative LC-MS chromatograms of the synthesized oligonucleotides X_n. The insets show mass spectra of highlighted peak and associated anions assignation.

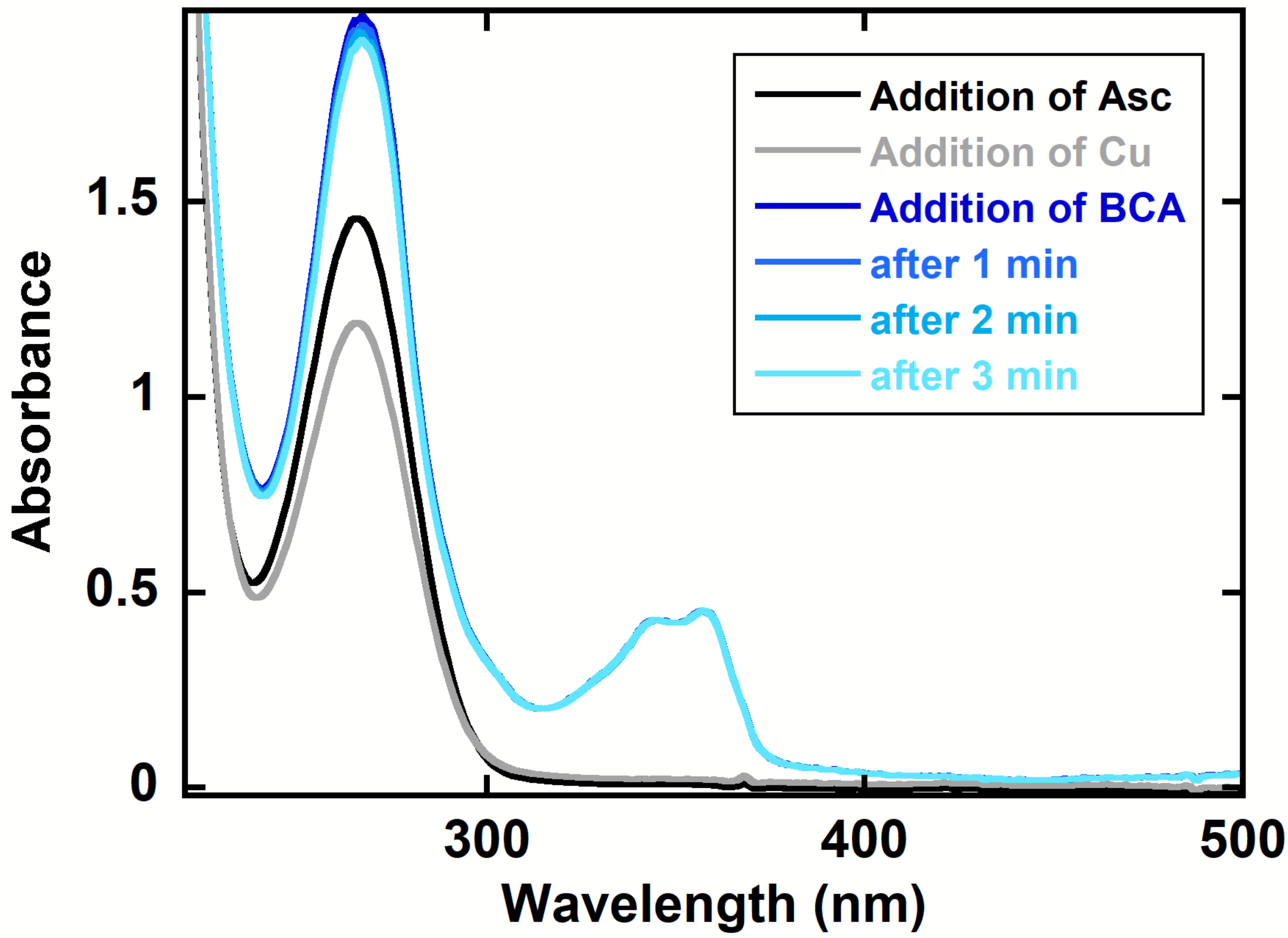

Figure S2. UV-visible spectra of Asc recorded over time, with Cu^{2+} added after 60 s and BCA added after 120 s. Conditions: [HEPES] = 50 mM pH 7.4; [Asc] = 100 µM; [$CuSO_4$] = 10 µM; [BCA] = 22 µM.

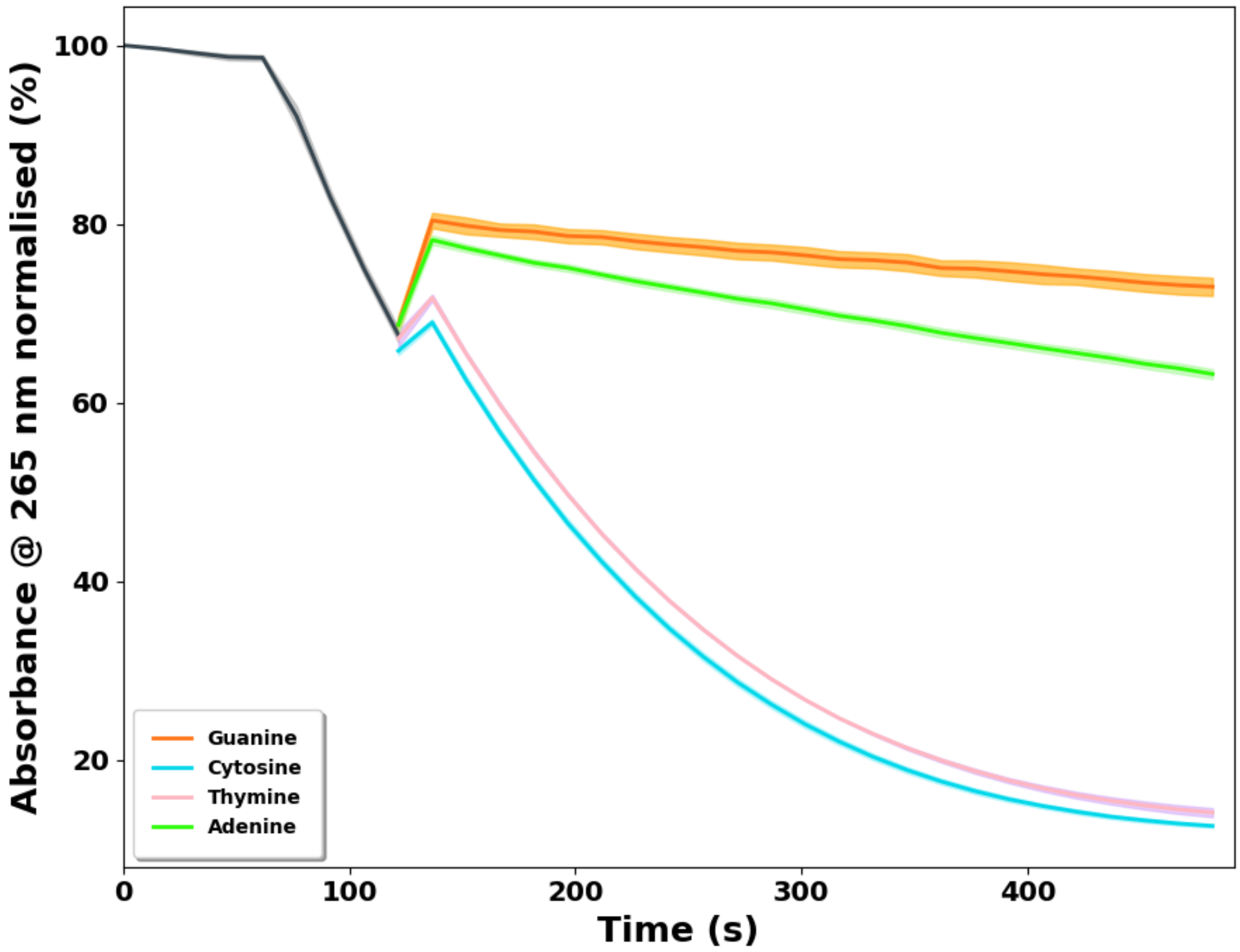

Figure S3. Normalized absorbance measured at λ = 265nm over time. Kinetic of ascorbate consumption in presence of Cu (added at 60 s) with addition of the four nucleobases (added at 120 s) : guanine, cytosine, thymine and adenine. Each bold line represents the average of three independent analyses (n = 3). The width of the shaded line corresponds to the standard deviation around the mean. Conditions: [HEPES] = 50 mM pH 7.4; [Asc] = 100 µM; [$CuSO_4$] = 10 µM; [nucleobases] = 20 µM.

A

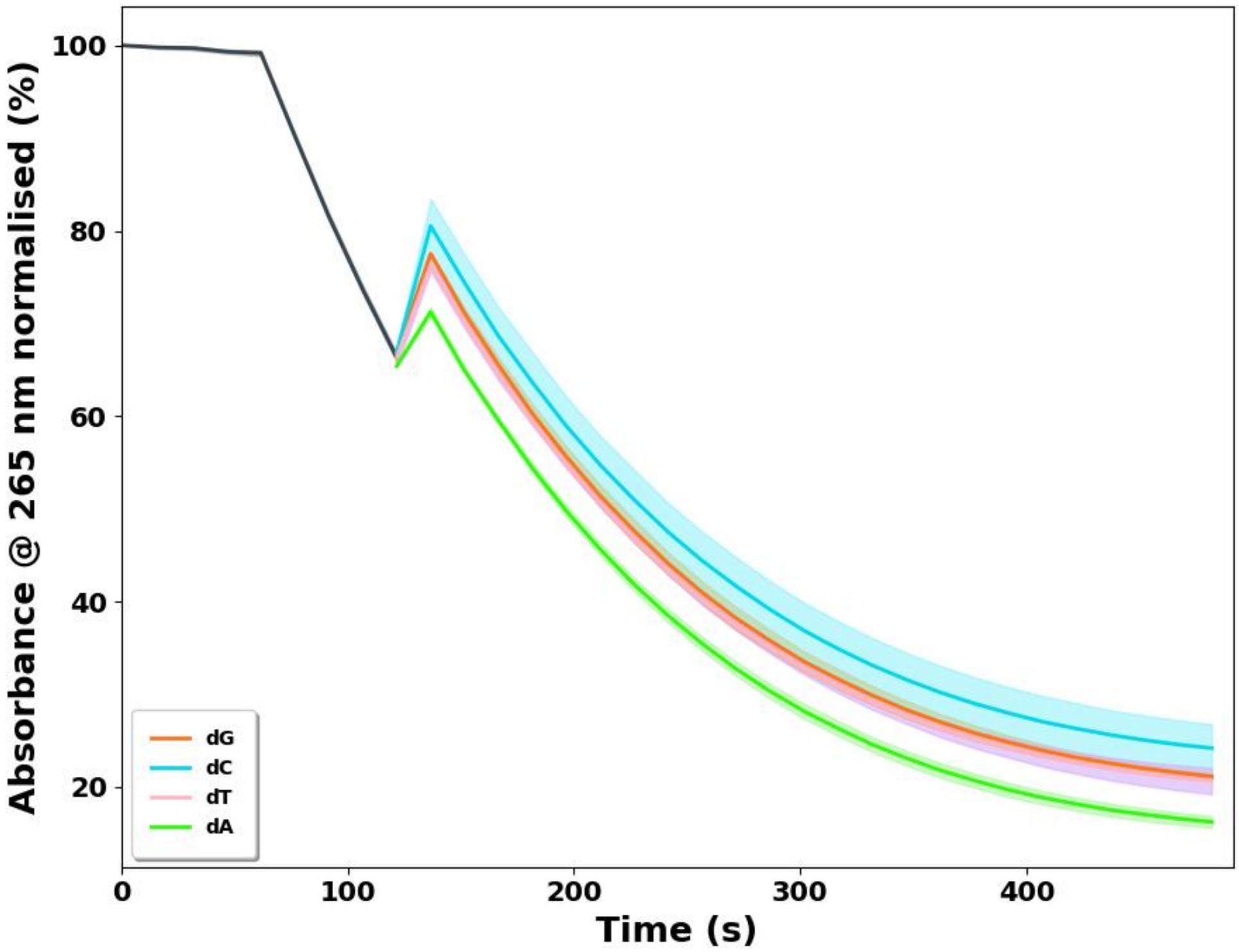

B

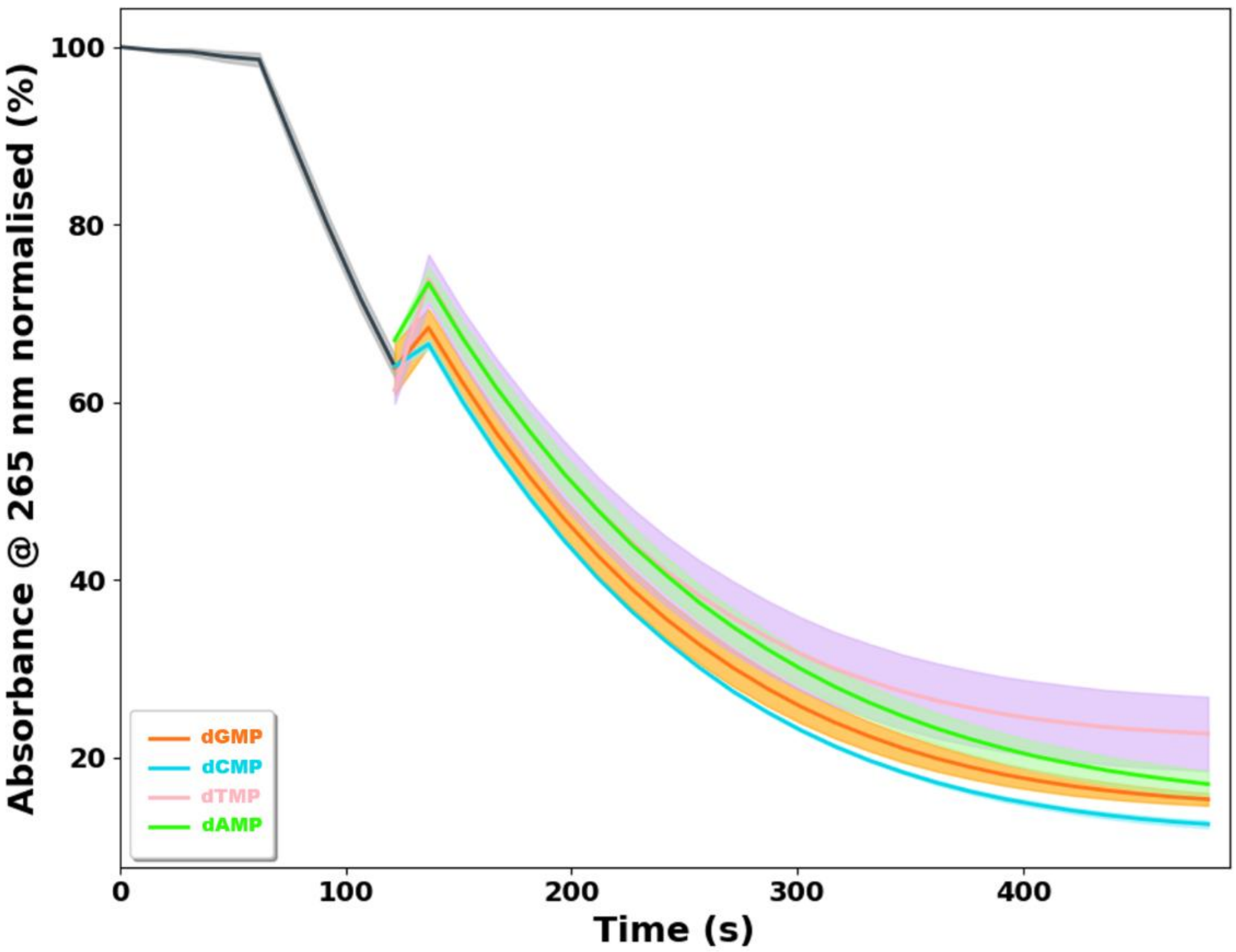

Figure S4. Normalized absorbance measured at λ = 265nm over time. Kinetic of ascorbate consumption in presence of Cu (added at 60 s) with addition of **A)** nucleosides : deoxyguanosine (dG), deoxycytidine (dC), deoxythymidine (dT) and deoxyadenosine (dA) or **B)** deoxynucleotides monophosphate : deoxyguanosine monophosphate (dGMP), deoxycytidine monophosphate (dCMP), deoxythymidine monophosphate (dTMP) and deoxyadenosine monophosphate (dAMP) added at 120 s. Each bold line represents the average of three independent analyses (n = 3). The width of the shaded line corresponds to the standard deviation around the mean. Conditions: [HEPES] = 50 mM pH 7.4; [Asc] = 100 µM; [$CuSO_4$] = 10 µM; [nucleosides] = [nucleotides] = 20 µM.

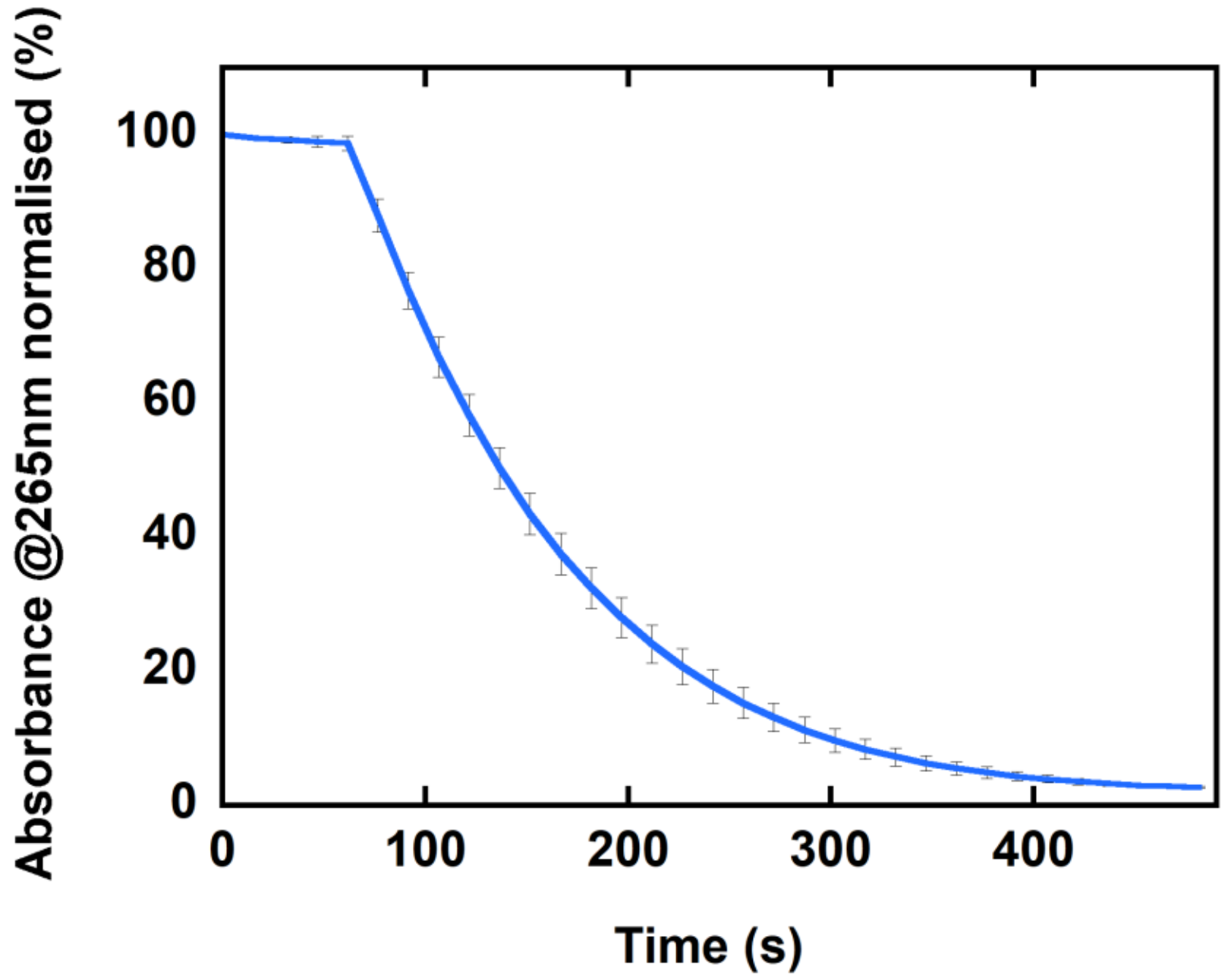

Figure S5. Normalized absorbance measured at λ = 265nm over time. Kinetic of ascorbate consumption in presence of Cu (added at 60 s) with addition of glycine at 120 s. Conditions: [HEPES] = 50 mM pH 7.4; [Asc] = 100 µM; [$CuSO_4$] = 10 µM; [Glycine] = 20 µM.

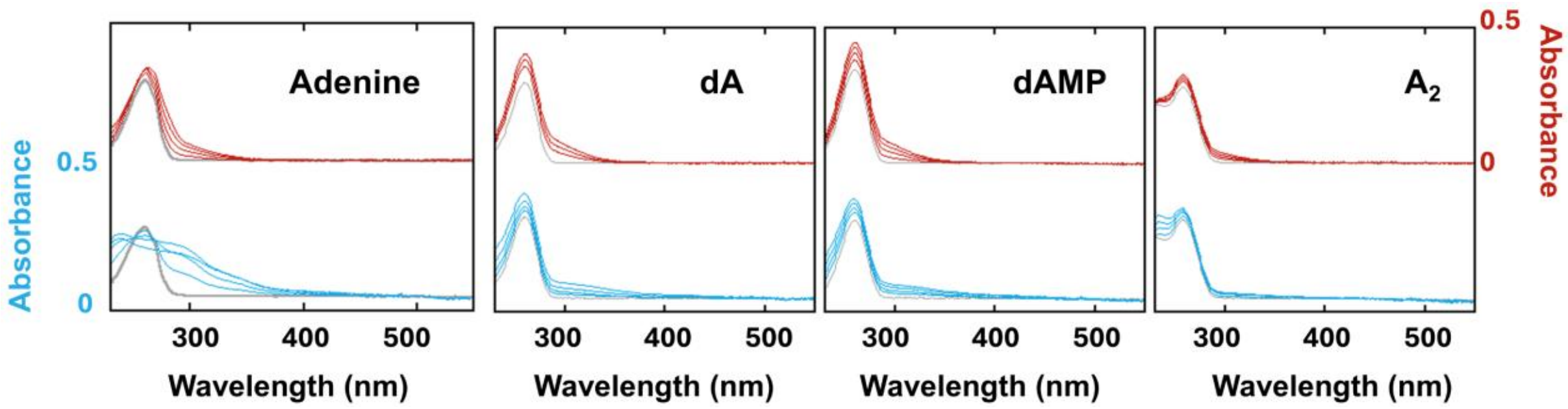

Figure S6. UV spectra of successive addition of Cu^{2+} (scale on right axis) and Cu^{+} (scale on left axis) of (from left to right) adenine, dA, dAMP, A_2 and A_6.Conditions: [HEPES] = 50 mM pH 7.4; [adenine] = 20 µM; [dA] = 20 µM; [dAMP] = 20 µM; [A_2] = 10 µM; [A_6] = 5 µM. Progressive addition of Cu^{2+} and Cu^{+} to reach a final molar ratio of 2.5.

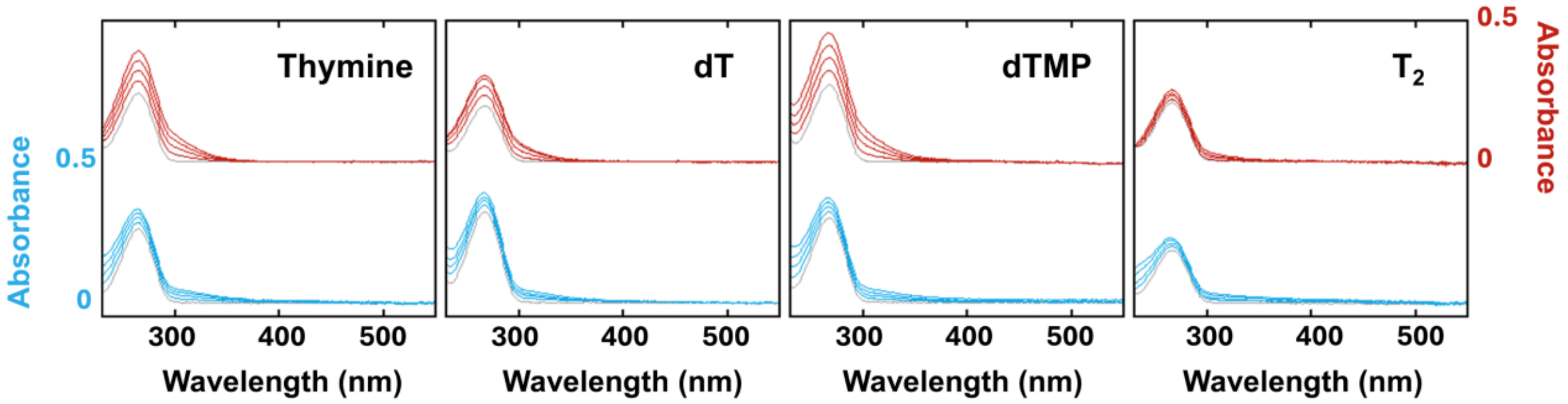

Figure S7. UV spectra of successive addition of Cu^{2+} (scale on right axis) and Cu^{+} (scale on left axis) of (from left to right) thymine, dT, dTMP and T_2.Conditions: [HEPES] = 50 mM pH 7.4; [thymine] = 30 µM; [dT] = 20 µM; [dTMP] = 30 µM; [T_2] = 10 µM. Progressive addition of Cu^{2+} and Cu^{+} to reach a final molar ratio of 2.5.

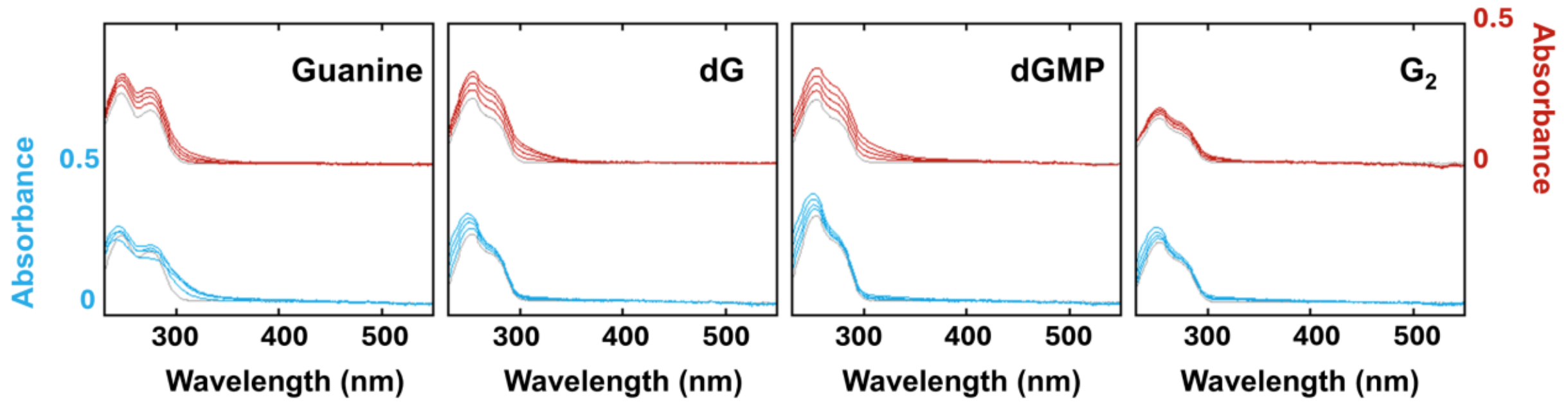

Figure S8. UV spectra of successive addition of Cu^{2+} (scale on right axis) and Cu^{+} (scale on left axis) of (from left to right) guanine, dG, dGMP and G_2.Conditions: [HEPES] = 50 mM pH 7.4; [guanine] = 20 µM; [dG] = 20 µM; [dGMP] = 20 µM; [G_2] = 8 µM. Progressive addition of Cu^{2+} and Cu^{+} to reach a final molar ratio of 2.5.

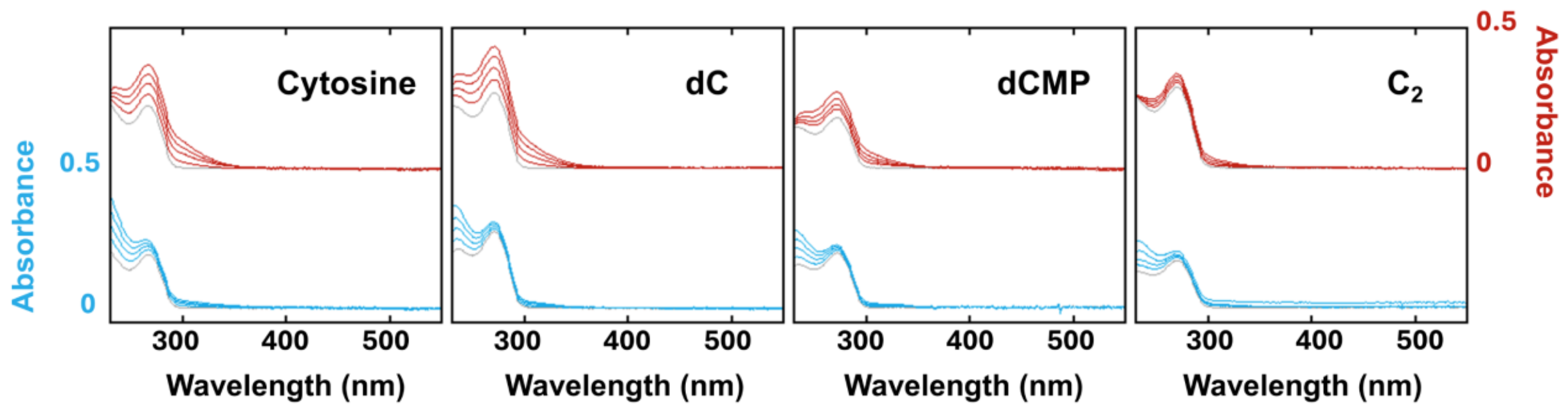

Figure S9. UV spectra of successive addition of Cu^{2+} (scale on right axis) and Cu^{+} (scale on left axis) of (from left to right) cytosine, dC, dCMP and C_2.Conditions: [HEPES] = 50 mM pH 7.4; [cytosine] = 30 µM; [dC] = 30 µM; [dCMP] = 20 µM; [C_2] = 12 µM. Progressive addition of Cu^{2+} and Cu^{+} to reach a final molar ratio of 2.5.

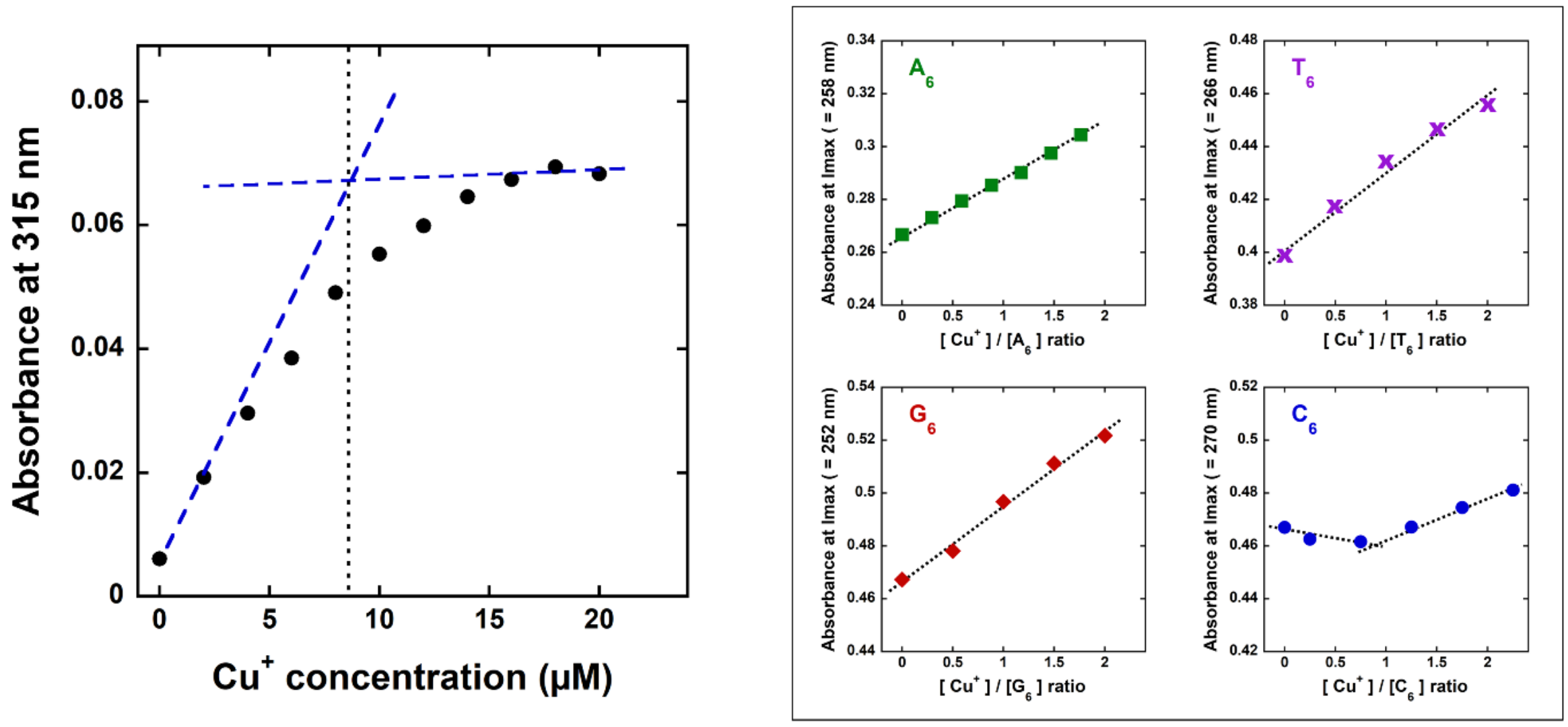

Figure S10. Cu^+ titration on C_6 monitored at 315 nm (left) and on X_6 monitored at their λ_{max} (right). The Cu^+ titration on C_6 show the formation of a 1:1 Cu^+ : C_6 complex. Based on this data of direct titration, C_6 affinity for Cu^+ was determined (log K = 6.3). Calculation details are described in Experimental Details section. Conditions: [HEPES] = 50 mM pH 7.4; [C_6] = 8 µM; [T_6] = 6 µM; [A_6] = 3.5 µM; [G_6] = 6 µM; [Cu^+] = 0-20 µM.

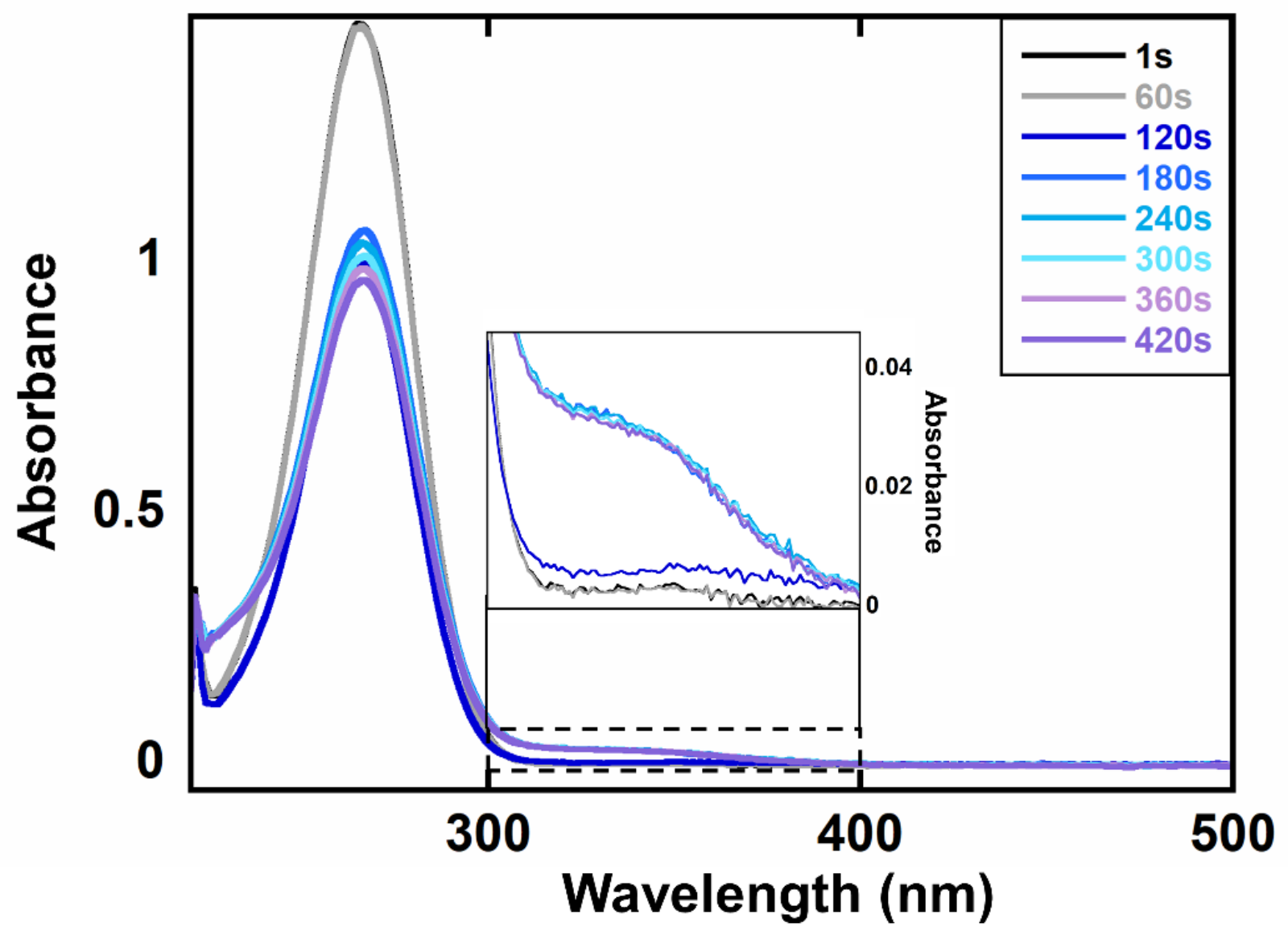

Figure S11 : UV–visible spectra of Asc recorded over time, with Cu^{2+} added after 60 s and C_6 added after 120 s. The inset shows a magnified view on y-axis of the 300–400 nm region. Condition: [HEPES] = 50 mM pH 7.4; [Asc] = 100 µM; [$CuSO_4$] = 10 µM; [C_6] = 3.33 µM.

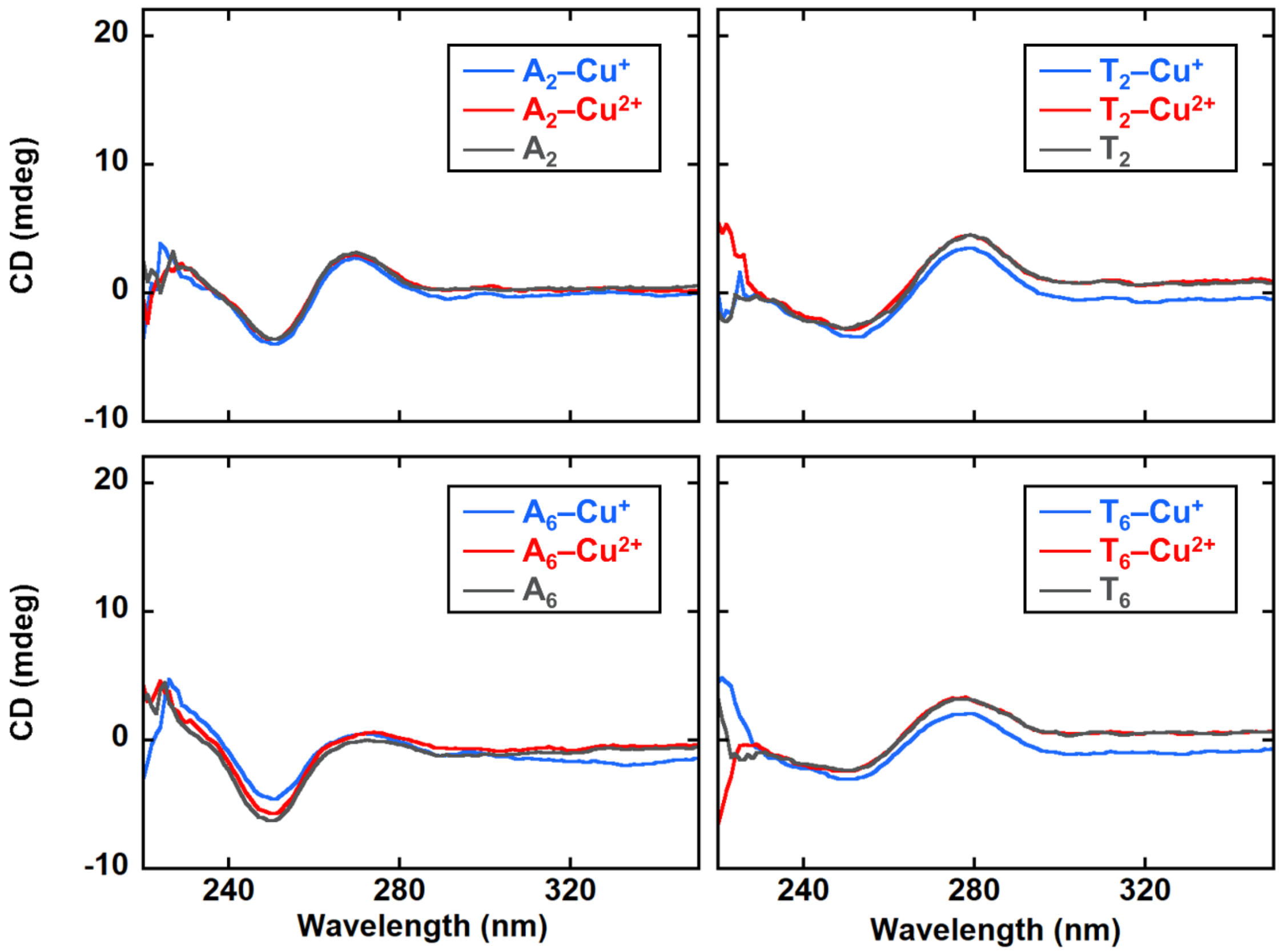

Figure S12. CD spectra of oligonucleotides in absence and in presence of Cu^+ and Cu^{2+} at stoichiometry 1:1. Conditions: [HEPES] = 50 mM pH 7.4; [A_2] = [Cu^+] = [Cu^{2+}] = 11 µM (top left); [A_6] = [Cu^+] = [Cu^{2+}] = 4 µM (bottom left); [T_2] = [Cu^+] = [Cu^{2+}] = 18 µM (top right) ; [T_6] = [Cu^+] = [Cu^{2+}] = 6 µM (bottom right).

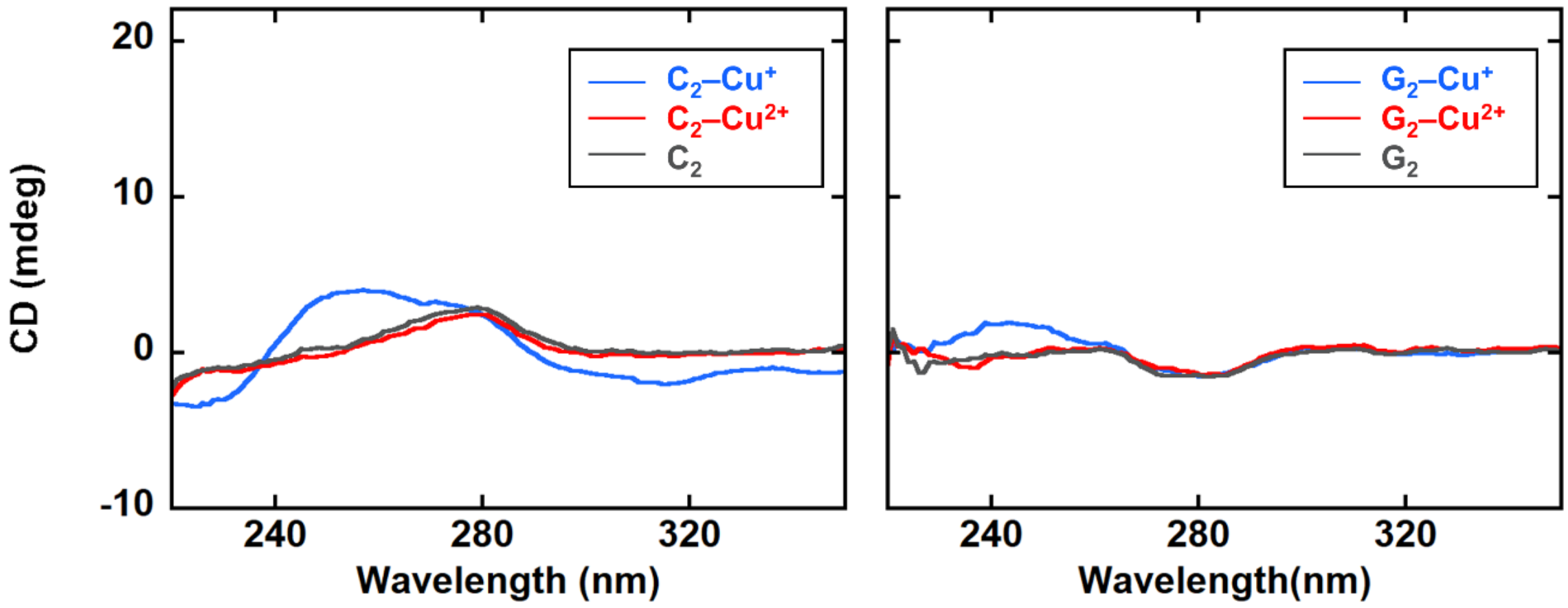

Figure S13. CD spectra of oligonucleotides in absence and in presence of Cu^{+} and Cu^{2+} at stoichiometry 1:1. Conditions: [HEPES] = 50 mM pH 7.4; [C_2] = [Cu^{+}] = [Cu^{2+}] = 20 µM (left); [G_2] = [Cu^{+}] = [Cu^{2+}] = 14 µM (right).

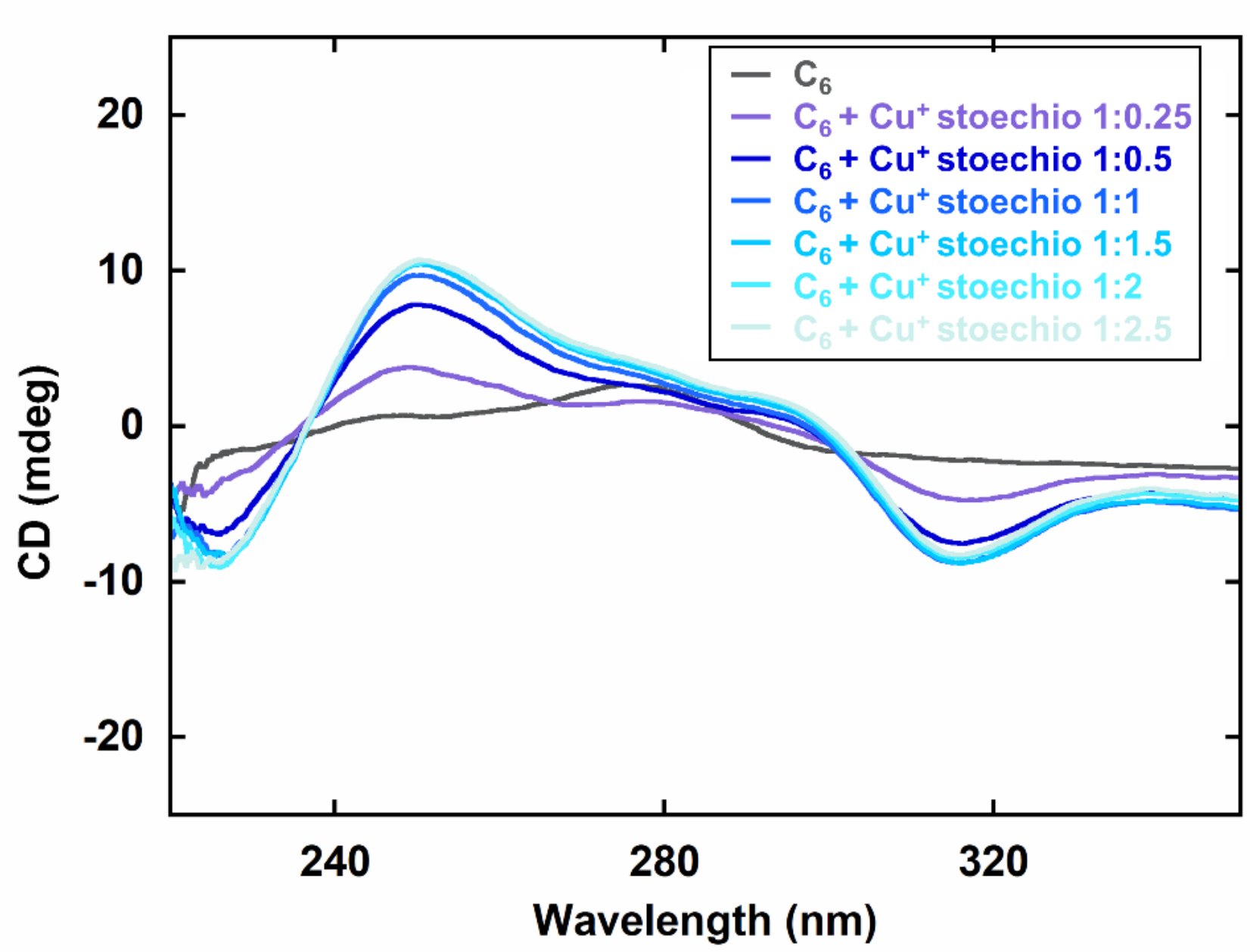

Figure S14. CD spectra of oligonucleotide C_6 in presence of Cu^{+} at different stoichiometry (shades of blue) 1 :0.25 ; 1 :0.5 ; 1:1; 1 :1.5; 1 :2 ; 1 :2.5. Conditions: [HEPES] = 50 mM pH 7.4; [C_6] = 8 µM; [Cu^{+}] = 0-20 µM

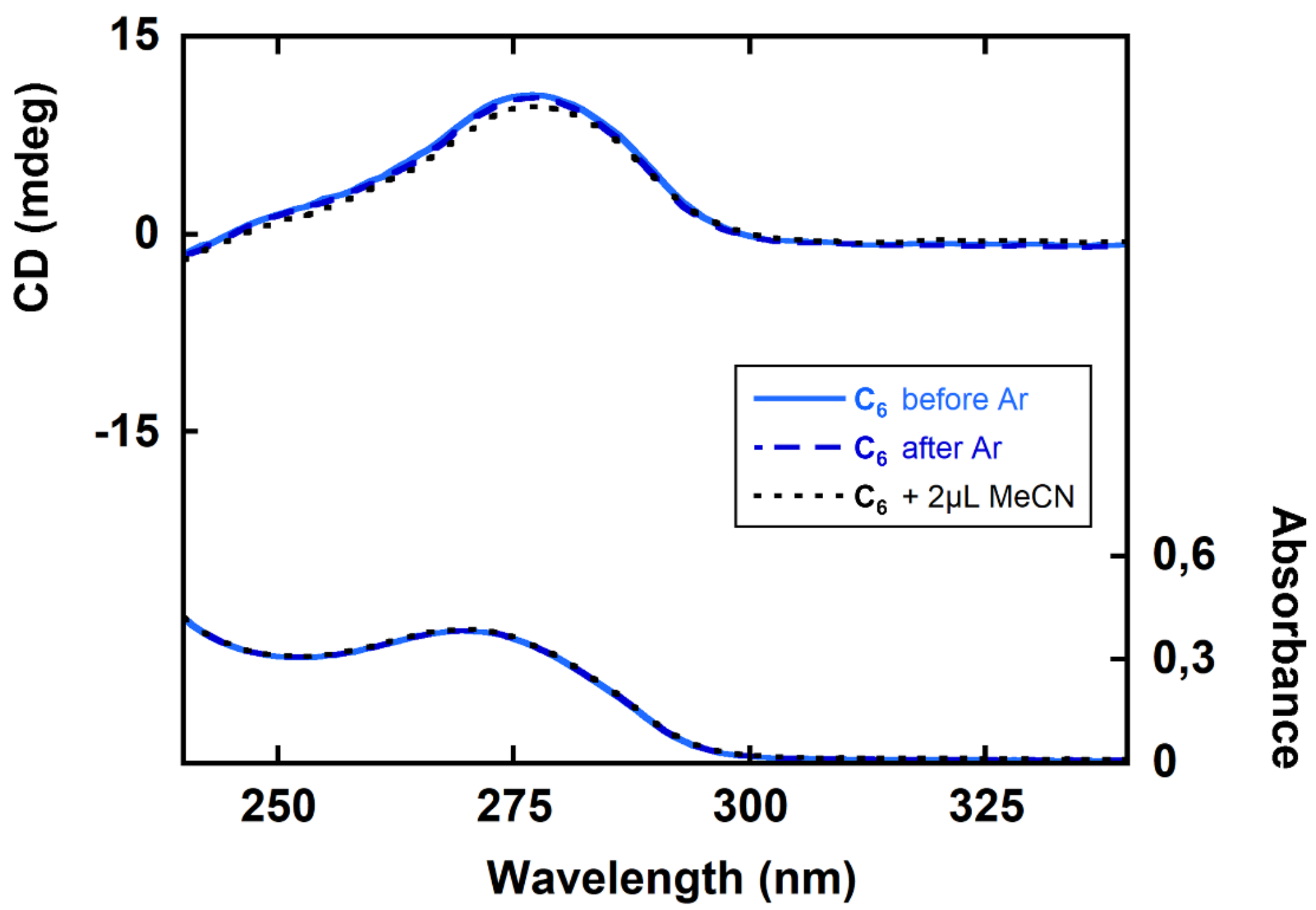

Figure S15. UV (scale on the right axis) and CD (scale on the left axis) spectra of oligonucleotide C_6 before (light blue line) and after (dark blue dashed line) oxygen depletion with Ar and in presence of acetonitrile (MeCN) (dark dashed line). Conditions : [HEPES] = 50 mM pH 7.4; [C_6] = 8 µM.

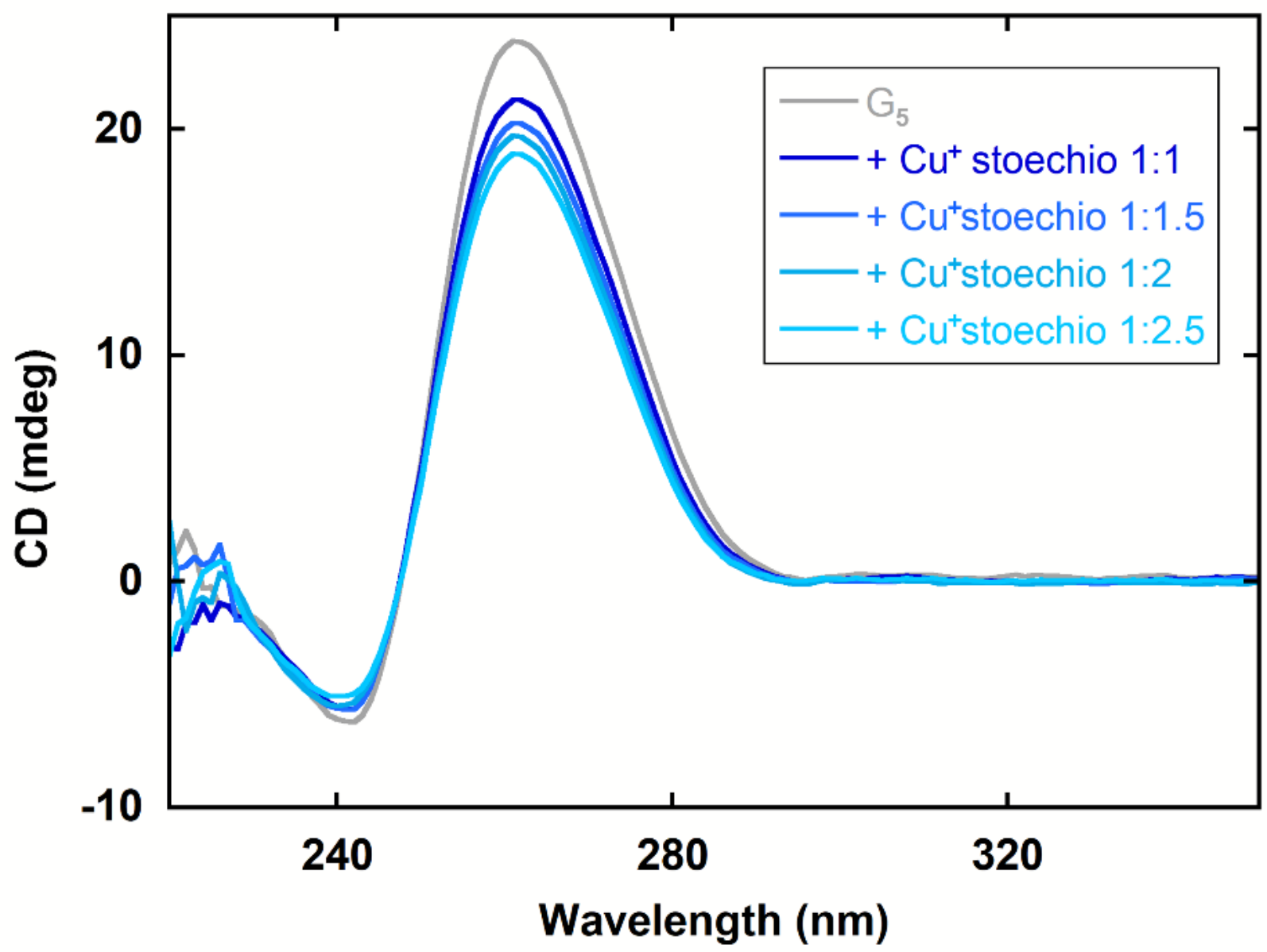

Figure S16. CD spectra of oligonucleotide G_5 in absence (grey) and in presence of Cu^+ at different stoichiometry (shades of blue) 1:1; 1 :1.5; 1 :2; 1 :2.5. Conditions: [HEPES] = 50 mM pH 7.4; [G_5] = 10 µM.

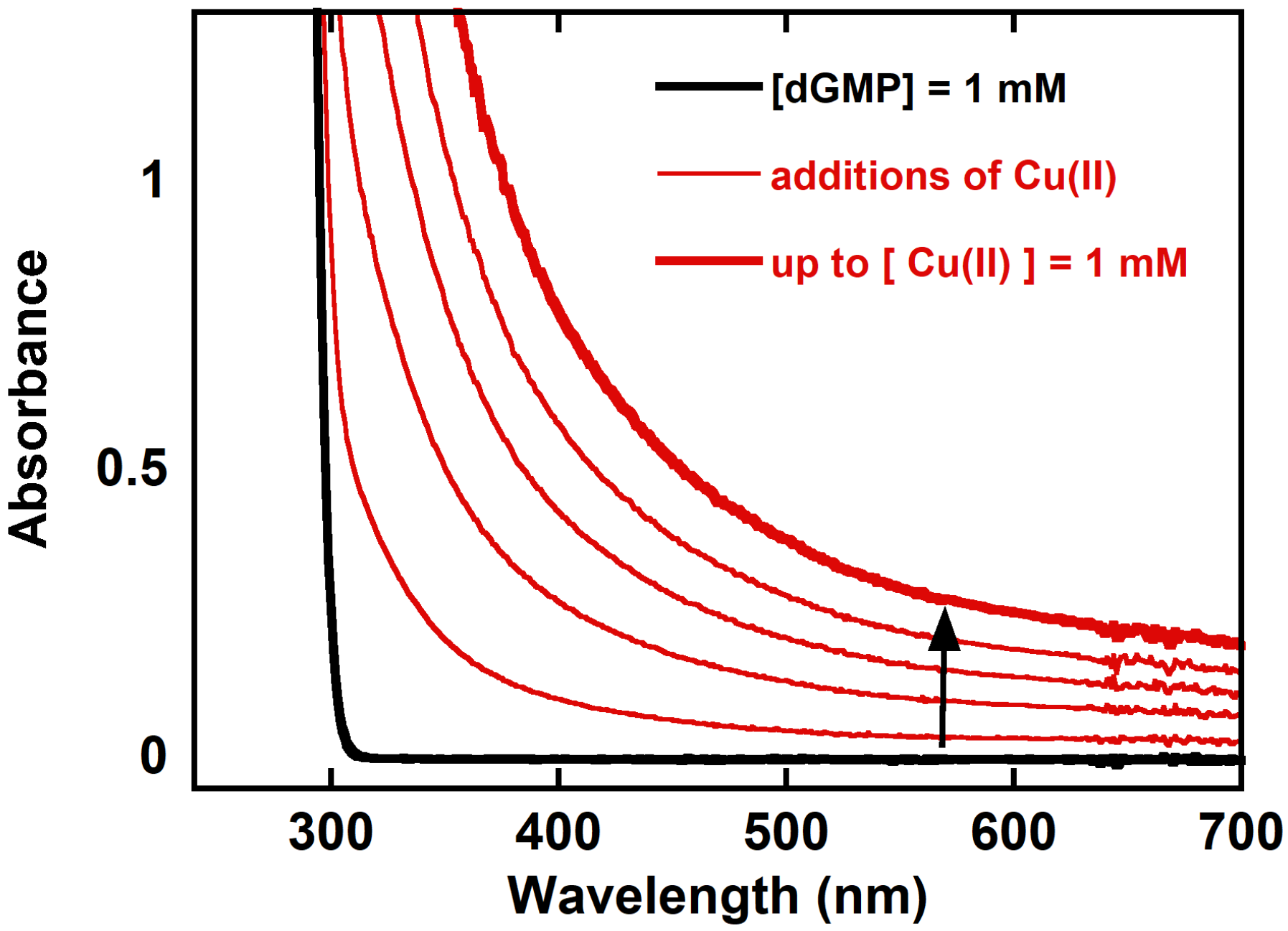

Figure S17. UV-Visible spectra of dGMP at 1 mM (bold black) and several additions of 0.2 mM Cu(II) (thin red) up to [Cu(II)] = 1 mM (bold red). The black arrow highlights the precipitation that occurs upon addition of Cu(II). This insolubility precludes the use of techniques requiring concentration on the order of hundreds of µM, such cyclic voltammetry, NMR or EPR. Condition: [HEPES] = 50 mM pH 7.4; [dGMP] = 1 mM; [Cu(II)] = 0-1 mM.
